# Setting a Comprehensive Bow‐Tie Framework for Disaster Risk Analysis of Mine Tailings Storage Facilities

**DOI:** 10.1111/risa.70137

**Published:** 2025-10-28

**Authors:** Rafaela Shinobe Massignan, Juliana Siqueira‐Gay, Luis Enrique Sánchez

**Affiliations:** ^1^ Department of Mining and Petroleum Engineering University of São Paulo São Paulo Brazil; ^2^ Department of Hydraulics and Environmental Engineering University of São Paulo São Paulo Brazil

**Keywords:** disaster risk reduction, dry‐stack facility, exposure, tailings dam, vulnerability

## Abstract

Disasters caused by tailings storage facilities (TSFs) have highlighted the complexity of safely managing mine tailings and the extension of consequences over time and throughout the tailings runoff. Investigations commissioned by mining companies following major failures in Mariana and Brumadinho, Brazil, primarily focused on immediate technical causes and hazards. However, for effective disaster risk reduction, the integration of technical, environmental, and social factors is needed to comprehensively address the complexity of risk management. Bow‐tie models can be used for TSF's disaster analysis, as they consider causes, consequences, and preventive and mitigation controls. Here, an adapted bow‐tie framework for TSF's disaster risk analysis is proposed to systematize the identification of threats and consequences and address the four disaster risk dimensions: hazard, exposure, vulnerability, and capacity. The framework was applied to the *Pontal* TSF, Brazil, using publicly available information, revealing gaps in the risk management, such as the lack of identification of social vulnerabilities. Our framework highlights the importance of reducing TSF's disaster risks through all dimensions and engaging multiple stakeholders. Although TSF stability control is primordial and irreplaceable, alone it is insufficient for effective disaster risk reduction.

## 1 Introduction

Failures of mine tailings storage facilities (TSFs) have been recorded since 1915 (CSP2 2022), highlighting the extension of the consequences to the environment, people, livelihoods, and economy, as well as the gaps and complexity in safely managing mine tailings. The deadliest failure registered in the database of the Center of Science for Public Participation (CSP2 2022) is the Sgurigrad disaster in 1966 in Bulgaria, resulting in 488 fatalities. Recent disasters in Brazil—the Fundão (Mariana) and Córrego do Feijão (Brumadinho) catastrophes, in 2015 and 2019, respectively—drew international attention to major gaps in risk disclosure and management (Owen et al. 2020). The failure in Mariana caused 19 fatalities, whereas in Brumadinho, 272 people perished. Both disasters polluted important rivers and compromised water supply, wildlife habitats, and livelihoods and displaced people. Their technical investigations pointed out that the liquefaction flowslides were caused by saturated conditions and inefficient internal drainage close to the upstream dam crests (Morgenstern et al. 2016; Robertson et al. 2019). Considering only technical causes and immediate consequences was also suggested by previous academic studies for assessing a tailings dam's risk of failure (Salgueiro et al. 2008). Such an approach to risk analysis contradicts international recommendations on disaster risk reduction that acknowledge people's exposure and vulnerability and emergency preparedness (UNEP 2001; UNISDR 2015; IPCC 2023).

To comprehensively understand and prevent disasters, they must be framed beyond engineering immediate causes (Marais et al. 2024), unearthing organizational and regulatory factors (Rose et al. 2023), and exposure and vulnerability conditions (Kemp 2020). The importance of considering the hazard‐prone area is highlighted by the very definition of disaster adopted by the United Nations, as a disaster results from the interaction of hazards, exposure, vulnerability, and capacity, leading to human, material, economic, and environmental losses and impacts (UNGA 2016). Nonetheless, these guidelines for disaster risk analysis seem not to be comprehensively applied for tailings management. Workplace and process risks are usually analyzed separately from vulnerability assessments (Oliver‐Smith et al. 2016; Wisner et al. 2003) when applying standard risk assessment tools such as fault and event trees, hazard and operability study (HAZOP), and failure mode effects and criticality analysis (FMECA). Turner et al. (2003) developed a conceptual vulnerability framework considering a human–environment system, which applies to people, organizations, and ecosystems. Nevertheless, this model does not consider the vulnerability of assets, and the hazard is not specified.

Here, we seek to show that to comprehensively analyze disaster risks, bow‐tie models are suitable to illustrate the complexity of events, as they highlight not only independent pathways to a top event but also the importance of mitigation controls to reduce consequences (Hopkins 2012). Regarding TSFs, Chen et al. (2022) identified the causes of failure in four categories: human, environmental, facility, and management, for each life cycle stage: design, construction, and operation. The consequences were related to immediate environmental impacts, long‐lasting effects, and changes in ecosystem and social. To consider the event intensity, tailings volume release and distance travelled were added in a three‐dimensional risk matrix, with similar causes and consequences categories (Chen et al. 2023). However, both studies neglect the social and environmental vulnerability of the hazard‐prone area, whose understanding is paramount for risk reduction (UNISDR 2015; Kemp 2020).

The improvement of TSF's risk analysis is critical to avoid the reoccurrence of failures, which sum at least 376 since 1915 (CSP2 2022), and reduce the consequences of events. Moreover, this need is urgent, as the quantity and dimension of TSFs tend to increase with the intensification of tailings production due to critical minerals production (Valenta et al. 2023) and lower ore content reserves (Araya et al. 2021; Sánchez and Franks 2022). Considering that TSFs are permanent and climate change increases the frequency of extreme events (IPCC 2023), the failure and disaster risks tend to escalate.

Here, we propose a new approach to enhance multidisciplinary analysis of TSF's disasters by adapting a qualitative bow‐tie framework for considering the four dimensions of disaster risk—hazard, exposure, vulnerability, and capacity. We systematize the identification of threats and consequences and propose examples for control, mitigation, and recovery, based on literature and good practices guidance. The novelty of the approach is addressing exposure and vulnerability conditions of people, anthropic and natural environments, and considering long‐term consequences and recovery controls, as well as human threats.

Following this introduction, Section 2 presents the methodology, Section 3 explains the components of the adapted bow‐tie, and Section 4 proposes seven steps for applying the framework. For road testing the tool, it is applied to the *Pontal* TSF, a large structure in Minas Gerais (Section 5). We conclude by highlighting the importance of comprehensive disaster risk analysis.

## 2 Methods

**FIGURE 1:**
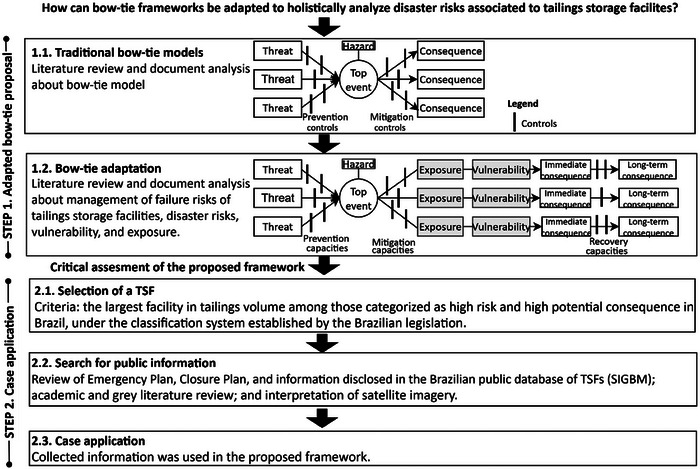
Research methodology. TSF, tailings storage facility.

This research is grounded on a review of scientific and technical literature, document analysis, and a case application, organized in two major steps (Figure 1).

### 2.1 Step 1. Bow‐Tie Adaptation

First, the components and methodology of traditional bow‐tie models were analyzed using the Australian Government's Risk Management handbook for the mining industry (Australia 2016) as the primary source.

In the sequence, the scope of the proposed framework was limited to systemize the identification of (i) threats and preventive controls; (ii) factors of the hazard‐prone area that influence disaster's risks and mitigation controls; and (iii) consequences of TSF's disasters and recovery controls. For each theme, specific literature review was conducted, as follows.

To identify technical and environmental threats and preventive controls, literature was searched on Scopus using combinations of the keywords “tailings storage facility,” “causes of failure,” and “risk management,” in March and April 2023. Three types of facilities that impose failure risk were considered: tailings dams, in‐pit, and dry‐stack disposal. Moreover, other references of TSF management were used: the book “Planning, Design, and Analysis of Tailings Dams” (Vick 1990), the “Guide to the Management of Tailings Facilities” (MAC 2021), and the “Safety First Guidelines for Responsible Mine Tailings Management” (Morrill et al. 2022). Organizational threats and controls were also identified in the “Global Industry Standard on Tailings Management” (GISTM) (GTR 2020) and the compendium of articles “Towards Zero Harm” jointly launched, as well as Hopkins and Kemp (2021), who discuss the GISTM development.

Two factors of the hazard‐prone area were considered: exposure and vulnerability, according to the concept of disaster risks (UNGA 2016) of social conditions, anthropic environment, and ecosystems. For social conditions, the Pressure and Response (PAR) model of vulnerability (Wisner et al. 2004; Oliver‐Smith et al. 2016) was analyzed. For the anthropic environment, the guidelines of UNESCO for disaster risk reduction for cultural heritages were assessed. In addition, the concept of “critical habitats” (IFC 2012) was considered for ecosystem vulnerability. Mitigation controls were analyzed in the respective literature regarding emergency preparedness and resilience increase.

The third literature review aimed to identify consequences and recovery controls of disasters of TSFs through analyzing literature about the outcomes of the Fundão dam and Córrego do Feijão mine. The searches were made in Scopus using the keywords “doce river” and “brumadinho,” respectively, in May 2025.

### 2.2 Step 2. Case Application

After concluding the framework, we selected the Pontal system to conduct a case study. In July 2024, it was the TSF with the largest tailings volume—227.9 Mm^3^—among those categorized as high risk and high potential consequence in Brazil (ANM 2024), according to the national classification system (ANM 2022). Information about the structure, hazard‐prone area, and controls was searched in the public database SIGBM of Brazilian tailings dams (ANM 2024), the emergency plan of Pontal, and its closure projects. Academic and grey literature and satellite images were consulted for environmental conditions identification, as listed in Supporting Information A. On the basis of information collected in July 2024, we identified threats and consequences. Preventive barriers were only analyzed if the respective threat was identified and if applicable to closure, Pontal's current life cycle stage. Information not found in the publicly available documents was considered as non‐existent in the bow‐tie framework.

## 3 The Bow‐Tie Adaptation

Traditional bow‐tie models are the combinations of event and fault trees, identifying causes and consequences of a top event, and prevention and mitigation controls. The latter are instruments or human actions that by themselves prevent or mitigate events (Australia 2016), which in disaster terminology relate to capacity—strengths, attributes, and resources to manage and reduce disaster risks and strengthen resilience (UNGA 2016). Bow‐ties may be developed through seven steps (Australia 2016): (i) description of the unwanted event; (ii) scope determination; (iii) identification of threats; (iv) consequences; (v) prevention and mitigation controls; (vi) failure modes for important controls; and (vii) determination of assurance requirements for controls to remain available and responsive over time. Regarding Step (iii), threats, also named risk factors, by themselves may not pose a significant risk, but a large number of threats impose a greater risk (Hopkins 2011). Although this methodology is comprehensive, it is general for the mining sector, and identification steps in bow‐tie models still lack systematization to prevent hidden subjects (Aust and Pons 2020; Zhang et al. 2018).

Therefore, adaptation for analyzing disaster risks of TSFs was made, as explained in the sequence.

### 3.1 New Topics of Vulnerability and Exposure on the Bow‐Tie Diagram

Acknowledging exposure and vulnerability conditions of people, ecosystems, and anthropic environments in mitigation controls could reduce the most disastrous ruptures. Considering the hazard‐prone area is relevant because, even though all the factors that influence a TSF failure are considered, still the probability is not none, due to uncertainties related to calculation models, quantity and quality of input data (Cornell and Jackson 2013), and climate change events (IPCC 2023).

Exposure regards the location of people, assets, infrastructure, livelihoods, and ecosystems in hazard‐prone areas (Turner et al. 2003; Oliver‐Smith et al. 2016; UNGA 2016), which is identified in inundation maps of TSFs (MAC 2021). In these documents, exposure should not be limited to inundation areas but extended to any community, flora, or fauna whose livelihood or ecosystem will be affected. Therefore, mapping the exposure conditions indicates probable consequences, such as deaths, injuries, losses, damages, and secondary events caused by the exposure of hazardous installations, such as water dams (Australia 2016).

**FIGURE 2:**
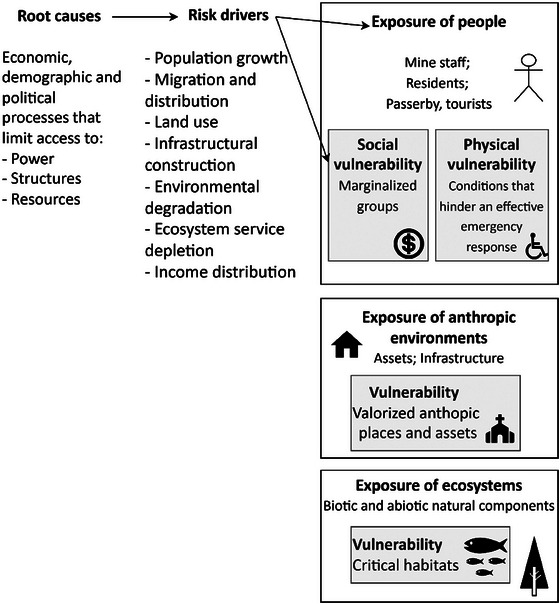
Exposure and vulnerability conditions of the hazard‐prone area. *Source*: Adapted from: Wisner et al. (2004) and Oliver‐Smith et al. (2016).

Although exposure indicates potential affected communities and environments, their risk is not the same due to individualized vulnerabilities. This condition intensifies the degree of consequences (Roque et al. 2023), reducing the resilience, that is, the ability to resist, adapt to, and recover in a timely and efficient manner (UNGA 2016). In the sequence, the vulnerability of people, anthropic environments, ecosystems, and mitigation barriers are described (Figure 2).

#### 3.1.1 Social and Physical Vulnerability of People

Exposure and vulnerability of people are socially constructed by root causes (Wisner et al. 2004), which are manifested by risk drivers (UNISDR 2015), leading to changes in population growth, land use, ecosystem service depletion, and income distribution (Oliver‐Smith et al. 2016). Vulnerability caused by risk drivers (Wisner et al. 2004) are classified in this article as social vulnerability. For instance, after the Córrego do Feijão disaster, fisherman communities were more intensively affected due to the mortality of fishes, fishing restrictions, and unsatisfactory level of education (Guimarães et al. 2024). In addition, a second classification of human vulnerability, named “physical vulnerability,” was considered, which refers to the biological and situational conditions that hinder an effective emergency response. This includes limited mobility among elderly individuals, children, and people with disabilities (Alvalá et al. 2019; Morrill et al. 2022), as well as proximity to the TSF without sufficient time to evacuate (Rose et al. 2023) and complexity to evacuate in facilities such as prisons, hospitals, and schools (Morrill et al. 2022).

Reducing exposure and vulnerability conditions minimizes disaster risks, although they depend on political decisions (Wisner et al. 2004), such as legal controls to restrict the hazard‐prone area (Oliver‐Smith et al. 2016). For social and physical vulnerability reduction, resilience should be strengthened by considering them in emergency plans (Alvalá et al. 2019) and increasing risk perception. Therefore, timely information of TSF failure risks and emergency response must be disclosed for exposed people (Owen et al. 2020), through in‐person communication and online initiatives (Massignan and Sánchez 2024). In addition, the emergency plan and evacuation simulations must be developed jointly with communities, civil defense, medical services, and other external organizations (UNEP 2001; GTR 2020; MAC 2021; Morrill et al. 2022). Pets, production, and wild animals should also be considered in emergency plans for faster evacuation and avoiding illegal reentrance in evacuated areas (Glassey et al. 2023). Moreover, for effective emergency response, alarms, warnings, and signs must be in place.

#### 3.1.2 Anthropic Environment

Exposure of anthropic environments—including buildings, transport infrastructure, mobile assets, croplands, and immaterial assets—is linked to the immediate and direct socioeconomic consequences of loss and damage. Consequently, leading to innumerous indirect outcomes, such as displacement, interruption of water supply, livelihood damage, and economic loss. Nonetheless, cultural heritages—including artifacts, monuments, buildings, and sites—are vulnerable, as their damage and loss interfere with social values, such as historic and aesthetic ones (UNESCO Institute for Statistics 2009). Their destruction could induce social consequences of loss of cultural activities and practices. Mitigation controls may include relocating heritages to outside the hazard‐prone area, constructing infrastructures to reduce the inundation area, and rescue plans. These controls should have minimal impact on the values and integrity of the heritage (UNESCO 2010).

#### 3.1.3 Ecosystem Vulnerability

Exposure of ecosystems, that is, biotic and abiotic natural components, might lead to immediate and direct consequences, such as flora and fauna mortality and water, soil, and air deterioration. Consequently, causing food chain contamination and further mortality in the long term. Intensified outcomes will disproportionately impact vulnerable ecosystems, which was related to “critical habitats” (IFC 2012). These host endangered, endemic, or migratory species; or are endangered and/or unique ecosystems; or provide relevant ecosystem services. Conservation measures include legal protection, although TSFs can be located within or close to protected areas worldwide (Aska et al. 2024; Kamino et al. 2020).

### 3.2 Considering Long‐Term Impacts and Recovery Controls

Disaster recovery aims to improve health, socioeconomic, and anthropic and natural environments, considering sustainability and resilience increase, by “building back better” (UNGA 2016). Effective recoveries reduce long‐term consequences, which manifest over months or years after the TSF failure. Social long‐term consequences include loss of social bonds within families and among friends, dependency of affected people for financial support and psychological effects, including the fear of new disasters caused by TSFs (Neri et al. 2021). These outcomes might induce an increase in alcohol, drugs, and criminality. Long‐term health consequences relate to inhalation of contaminated air and ingestion of contaminated water, crops, and animal protein (Murray et al. 1987), causing respiratory effects (Saraiva et al. 2024) and intensifying chronic diseases (Zhang et al. 2024). Examples of biophysical consequences are an increase in mosquito‐borne diseases and water and air contamination. For a successful recovery, communities must engage in decision‐making (Milanez et al. 2021) and have access to monitoring results (GTR 2020).

### 3.3 Addition of Human Threats to the Framework

**FIGURE 3:**
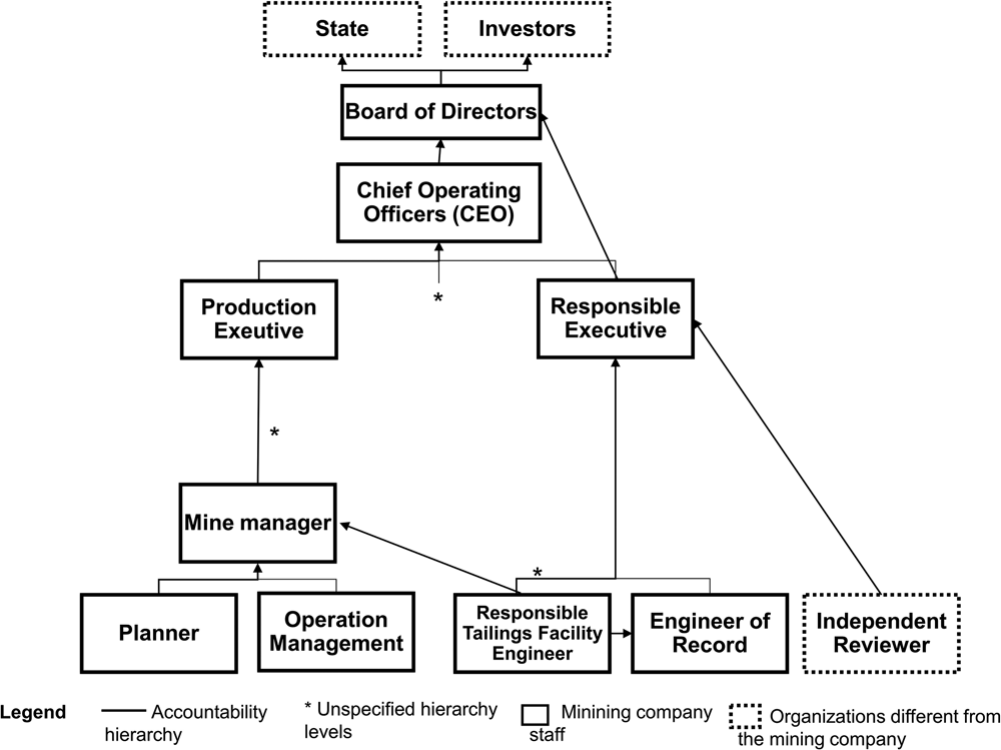
Hierarchy levels of accountability. *Source*: Adapted from Hopkins (2020), GTR (2020), and MAC (2021).

Many hazardous activities are conducted by companies, regulated by governmental agencies, and funded by financial institutions (Clarke 1999). Organizations are critical in creating and making decisions about risks. Therefore, in addition to the technical threats of TSFs, the functions of three key types of organizations were considered: governmental bodies, financial institutions, and mining companies (Figure 3).

Government, including environmental and mining agencies, and the processes of law making and enforcement, plays a key role to ensure licensing, auditing, and regulation of TSFs (Burritt and Christ 2021; Rose et al. 2023). These governmental roles should consider international good practices and law enforcement. Moreover, adequate resources must be allocated to regularly audit the facilities through on‐site inspection or satellite imagery analysis (Lumbroso et al. 2021) and drones. Solely relying on company‐reported monitoring information is insufficient, as critical risks may be underreported (Lanchotti 2023).

Financial institutions are also invaluable stakeholders of large‐scale mining companies, the investments of which should be constrained by the adoption of good practices of TSF management. Similarly, the International Finance Corporation demands that clients commit to eight environmental and social sustainability performance standards (IFC 2012). The Board of Directors of the mining company should account frequently to shareholders and not only after disasters (Hopkins 2020), enhancing disinvestment by troubling practices or events (Barrie et al. 2020). Therefore, financial institutions should hold the Board of Directors to account (Hopkins 2020), increasing transparency in risk information (Barrie et al. 2020).

The third organization is the mining company responsible for the TSF. Four aspects of this stakeholder are described: laws and good practices compliance, staff capacity, internal communication, and financial resources. The first should be ensured by an engineer‐of‐record (EoR) (this and other organizational levels are in Figure 3), who verifies if the project is in accordance with laws and good practices, as well as if the construction, operation, and closure attend to the project, good practices, and laws (GTR 2020; MAC 2021). The EoR must report to the responsible executive (GTR 2020), who argues for safety to the CEO and board of directors, in the same hierarchy levels as other executives (Hopkins 2020). Therefore, safety is discussed with the same priority as profit and production (Hopkins and Kemp 2021).

Furthermore, an independent reviewer—without economic conflict, friends, or family bonds with the company—should analyze the quality of risk management, governance effectiveness, and law compliance (GTR 2020; MAC 2021). Aiming to ensure the independence of this professional, the contract should be made through a regulatory agency (Hopkins and Kemp 2021), and the independent reviewer should emit a declaration of no conflict of interest (Morrill et al. 2022).

The second factor regards the qualification and experience of TSF safety employees to improve the facility management (GTR 2020). Therefore, not only should qualified staff be hired, but they should also be continuously qualified by offering training or motivating them to take new courses (GTR 2020; MAC 2021). Moreover, companies should develop a safety culture that motivates learning, early problem recognition, and internal communication (MAC 2021), which is the third factor of mining companies that influences the probability of failure.

Employers should be motivated to report TSF safety issues with protection for whistleblowers guaranteed, avoiding retaliation and discrimination (GTR 2020; MAC 2021; Morrill et al. 2022). Another threat of internal communication is the inexistence or inadequate change management. Hence, project and operation information must be updated and registered throughout the life cycle (GTR 2020; Valerius and Carvalho 2020; MAC 2021) to improve communication among current and future employers. Moreover, change management includes evaluation of the cumulative impact of changes in the TSF stability by the EoR and approval by the accountable executive (GTR 2020).

The fourth factor of mining companies is finance. Valorizing profit over safety compromises the adoption of safer technologies, for example, dry‐stack facilities, due to initial high costs. Hence, full cost accounting should be considered, including social and environmental externalities in the case of failure and costs throughout the life cycle (Morgenstern et al. 2015; Burrit and Christ 2021). This accounting should be reported to the responsible executive and the highest hierarchy levels—that is, the board of directors and CEO—as the two latter are responsible for the TSF (MAC 2021) and company image (Hopkins and Kemp 2021; Morrill et al. 2022). Another preventive control for profit valorization is considering safety in bonus mechanisms for employers (GTR 2020; Hopkins 2020; Hopkins and Kemp 2021).

### 3.4 Technical Threats of Failures of the TSF

The main triggers of TSF rupture are overtopping, foundation failure, piping, dynamic liquefaction, and excessively rapid dam construction (Azam and Li 2010; Baker et al. 2020). Nonetheless, there is rarely a single cause of rupture but a combination of risk factors, which are usually considered in bow‐tie models of TSFs (Chen et al. 2022). Here, technical threats are organized into tailings properties, facility characteristics, water management, and environmental conditions.

**FIGURE 4:**
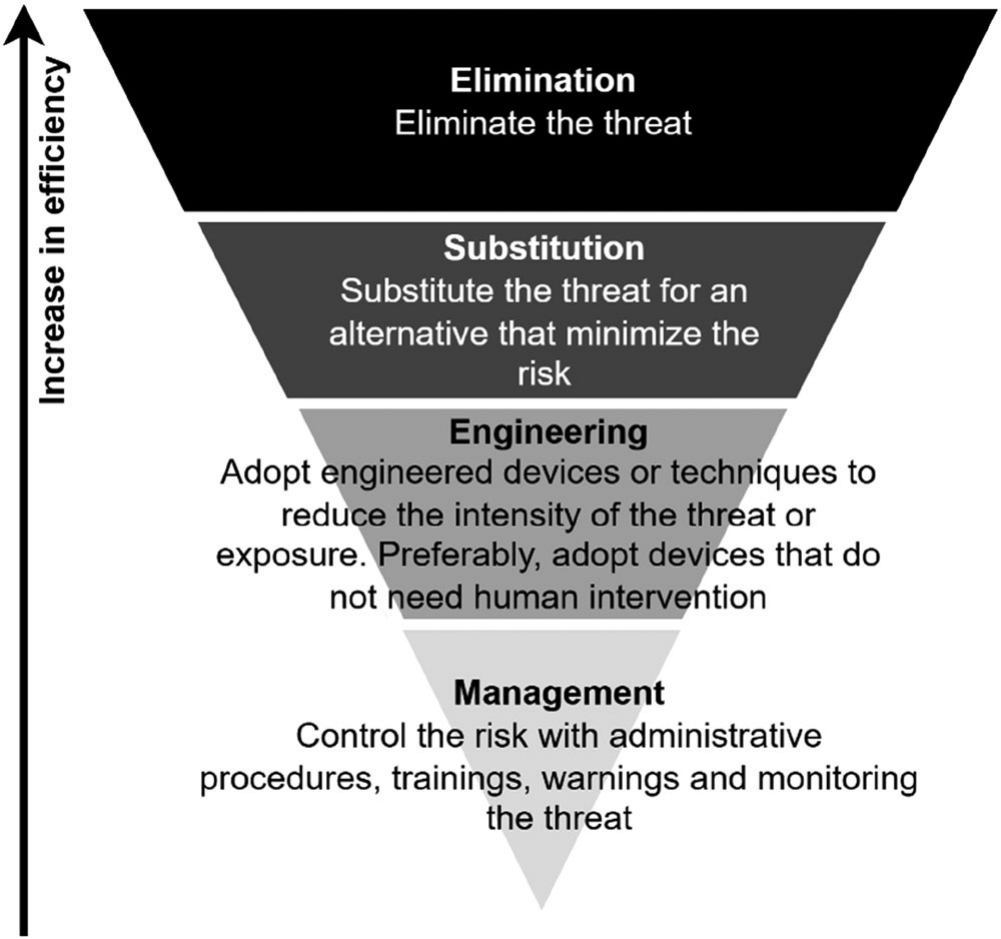
Control hierarchy in risk reduction. *Source*: Adapted from Australia (2016) and Chen et al. (2022).

Selection of controls should prioritize measures with higher efficiency, in the descending order: elimination of threats; substitution of threats for less risky factors; engineering measures that reduce threat intensity or exposure; and management of threats by preventing and controlling them (Australia 2016; Chen et al. 2022), as illustrated in Figure 4. Although eliminating the final disposal of tailings in TSFs is the control with the highest efficiency, alternatives are not always feasible. Therefore, substitution, management, and engineering preventive controls should be adopted and discussed jointly with the technical threats in the sequence.

#### 3.4.1 Tailings Properties

Tailings are the mineral beneficiation waste, composed of grains of multiple sizes and mineralogy, water, and chemical products, depending on the ore treatment. Therefore, their properties, such as geochemistry, grain size, and solid content, vary widely depending on the respective host rock and ore treatment methods. Regarding the first property, geochemistry influences the specific density of tailings, which should be considered in the vertical tensions calculation in the TSF. Moreover, geochemistry content may aggravate failure consequences, for example, in the case of acidic tailings containing heavy metals or toxic chemicals.

**FIGURE 5:**
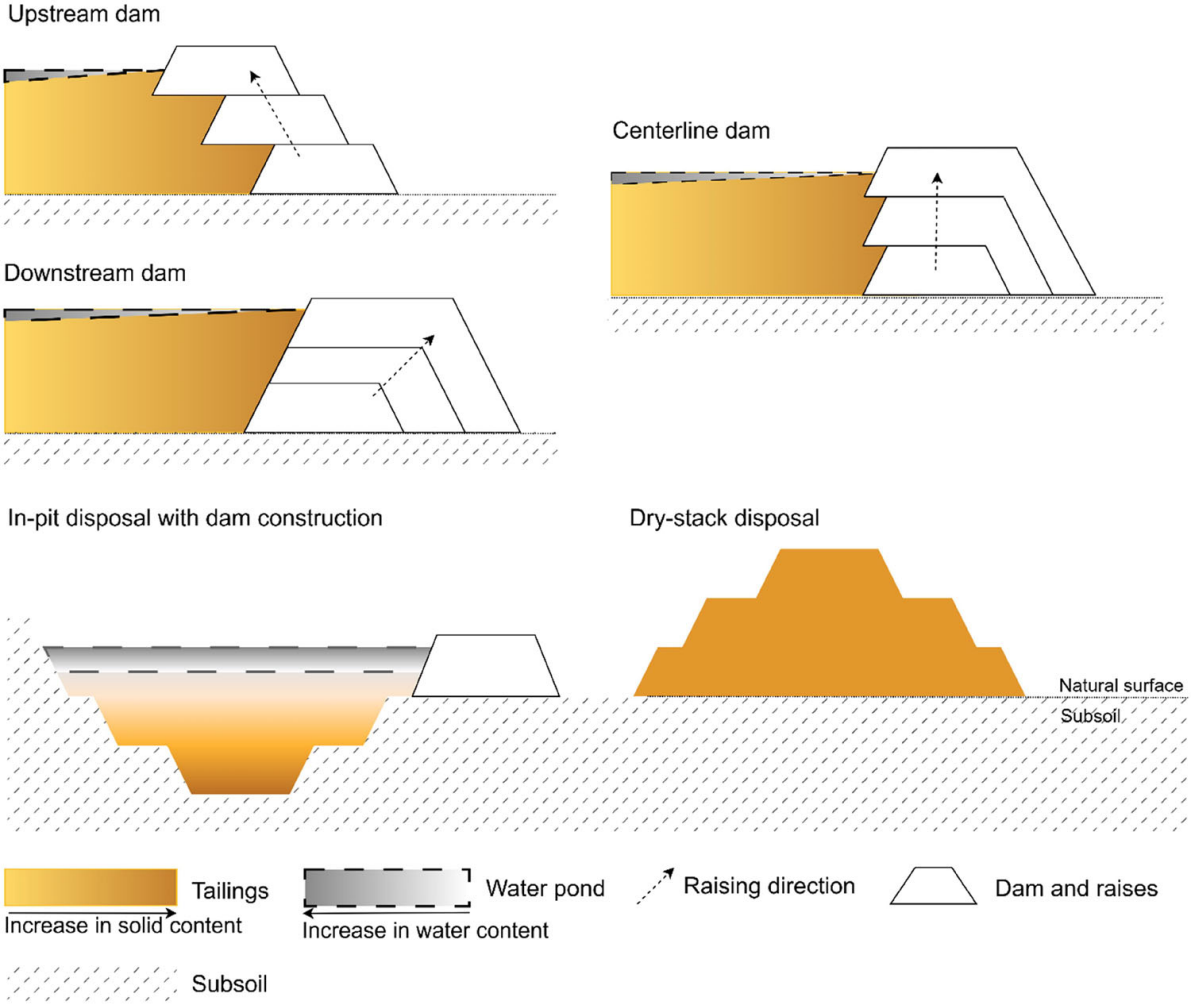
Comparative methods of tailings storage facilities construction.

Another tailings property is the grain size, which mainly consists of silt, clay, and sand, although some tailings may contain gravel fractions (Vick 1990), influencing permeability and consolidation. The smaller the grain size distribution, the lower the permeability (Vick 1990), hampering TSF internal drainage (Williams 2016). Hence, fine particles—that is, silt and clay—should be disposed of distantly from the dam, forming the beach region and a decant pond (Figure 5).

The third property is solid content, which varies according to how much water was used in the ore treatment and is reduced by thickening and filtering steps. Slurry tailings are pumpable and have the lowest solid content (Jewell et al. 2006); however, thickened and paste forms have higher solid content but still require a containment wall. The cake degree, on the other hand, is the most stable geotechnically, enabling consolidation into a dry‐stack facility, and is considered a best available technology (Morgenstern et al. 2015; Morrill et al. 2022).

In addition, co‐disposal of rock waste may reduce both the proportion of fine particles and water content (Burden and Wilson 2023), as rock waste usually is thicker than tailings and has no water addition. Therefore, mixing rock waste into tailings increases compressibility and permeability (Williams 2020).

#### 3.4.2 Facility Characteristics

Although all TSFs impose a risk of failure, dry‐stacking of cake‐grade tailings is the method with fewer instability reports (Franks et al. 2021), whereas upstream dam features the most (Azam and Li 2010; Piciullo et al. 2022). Because the beach region is the unconsolidated foundation of raises, upstream dams are more susceptible to liquefaction in seismic regions (Dobry and Alvarez 1967) and are not suitable for a large quantity of water disposal (Vick 1990). In contrast, centerline dams, although still improper for a large quantity of water, have acceptable seismic resistance (Vick 1990). It is an intermediate method between the upstream and downstream dams (Figure 5), considering that the latter is raised on the consolidated foundation of compacted tailings or rock waste. Hence, this method is more stable for seismic regions and water storage, less susceptible to liquefaction, and has no restriction for tailings size (Vick 1990).

Dams may be constructed in cross or side valleys (Ritcey 1989) or to raise the volume storage of an in‐pit disposal. For in‐pit disposal, stability of rock slopes must be evaluated in the preparation step, according to Cacciuttolo and Atencio (2023). Moreover, these authors highlight that subterranean water surges may hamper tailings consolidation, and superficial water should be pumped to enhance progressive closure.

Independent of the TSF type, rapid construction and raising may not ensure tailings consolidation, which is crucial to avoid instabilities. Furthermore, the design and other conditions of construction and operation influence the stability, for instance, by not following the approved design. In addition, steep slopes of embankments may induce instabilities on the TSF, decreasing its safety factor. Moreover, according to analyses of previous failures and instabilities of TSFs, their dimensions, for example, height and volume, are directly proportional to failure probability (Fourie 2009; Franks et al. 2021) and the volume released in a dam break (Piciullo et al. 2022).

Similarly, older facilities were more likely to report instabilities (Franks et al. 2021), highlighting the importance of proper closure and rehabilitation for chemical and physical stability (MAC 2021). These measures are often absent in inactive facilities—that is, TSFs that have ceased tailings discharge without rehabilitation—and in abandoned facilities. Thus, financial assurance (Morrill et al. 2022) and provision (Sánchez et al. 2014) should be guaranteed to ensure proper closure and prevent abandonment.

The geotechnical monitoring program, conducted throughout the life cycle, should be continued in the post‐closure to monitor probable failure modes (GTR 2020). Four main parameters are listed by Clarkson and Williams (2021): water level, pore pressure, seepage, and deformation. Visual inspection is highlighted to identify varied anomalies, such as accelerating displacements, tension cracks, seepages, and erosion features (Blight 2010).

#### 3.4.3 Water Management

Water management is a preventive control against hydrological threats and must be executed throughout the life cycle. Water is introduced in the TSF within the tailings, precipitations, runoff, subterranean surges, and, in the case of cross‐valley dams, natural drainage (Vick 1990; Blight 2010). Therefore, the quantity of water could be reduced by increasing the tailings solid content (Fourie 2006) or constructing dikes upstream of the TSF to diverge superficial runoffs and creek flows (Vick 1990). In case water balance is not properly considered, high pond level and high phreatic level may trigger overtopping and inadequate seepage, which could cause piping.

To improve water balance, conservative criteria must be considered in the planning stage, as Morrill et al. (2022) suggest the adoption of the largest probable flood in 10,000 years. Moreover, dam operation must observe a freeboard to prevent overtopping (Vick 1990); dams and raises should be impermeabilized, reducing the probability of dam seepage (Vick 1990); and a toe drainage system must also be considered to enhance water level control and prevent piping (Blight 2010).

In the operation stage, water may be reclaimed from the supernatant pond with pumping systems, penstocks, decant towers, and spillways (Blight 2010), which must be frequently unclogged (Valerius and Carvalho 2020). By reducing the pond or eliminating it, the phreatic water level is reduced, as well as saturated zones. Therefore, seepages are reduced and the liquefaction probability is decreased (Fourie 2006), which could be verified by monitoring water level and pore pressure with piezometers (Ritcey 1989; Blight 2010) and seepages with visual inspection (Blight 2010). If water surges are found, they should be properly investigated and managed (Valerius and Carvalho 2020).

#### 3.4.4 Environmental Conditions

Environmental conditions include the foundation, soil vibration, and climate‐related events. The first must be suitable for the final load of the facility, to avoid foundation failure. Therefore, the planning stage must include studies of foundation resistance and structural geology (Morgenstern et al. 2015) and prepare foundation treatment for the construction stage. Throughout the life cycle, horizontal and vertical deformations on the TSF should be monitored with inclinometers, topographical marks, and satellite or drone images (Valerius and Carvalho 2020) or modern automated stations.

Mining‐induced and natural seisms may also affect the TSF stability and trigger dynamic liquefaction (Vilcahuamán et al. 2018). Therefore, TSFs should be located away from rock blasting activities, upstream dams should be avoided in seismic regions, and vibrations should be monitored with seismographs. Additionally, failure of other technological structures, for example, water dams, upstream of the TSF may trigger a rupture.

The third environmental condition is climate‐related events, including not only meteorological events but also floods, landslides, and debris flow. Because these events tend to increase due to climate change (IPCC 2023), GTR (2020) and MAC (2021) highlight the need to consider a higher frequency of extreme events in the planning and management of the facility. Climate change resilience should be enhanced with adaptive management, which is based on monitoring data and updated information, reducing uncertainties throughout the life cycle (GTR 2020).

## 4 Proposal of a New Bow‐Tie and Case Application

### 4.1 Step‐by‐Step of Bow‐Tie Application

**FIGURE 6:**
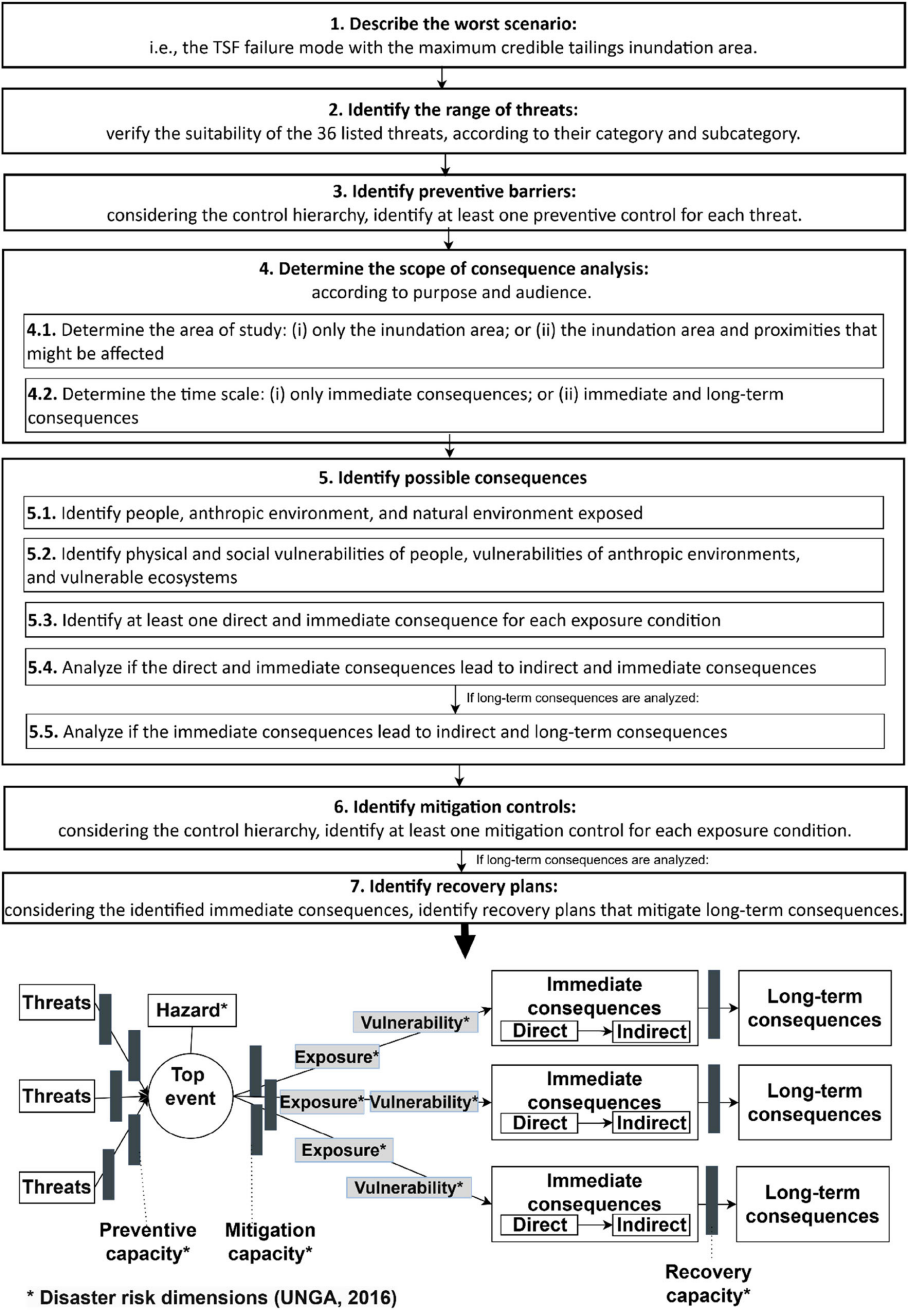
Proposed methodology for preparing a bow‐tie analysis of TSF's disasters. TSF, tailings storage facility.

**FIGURE 7:**
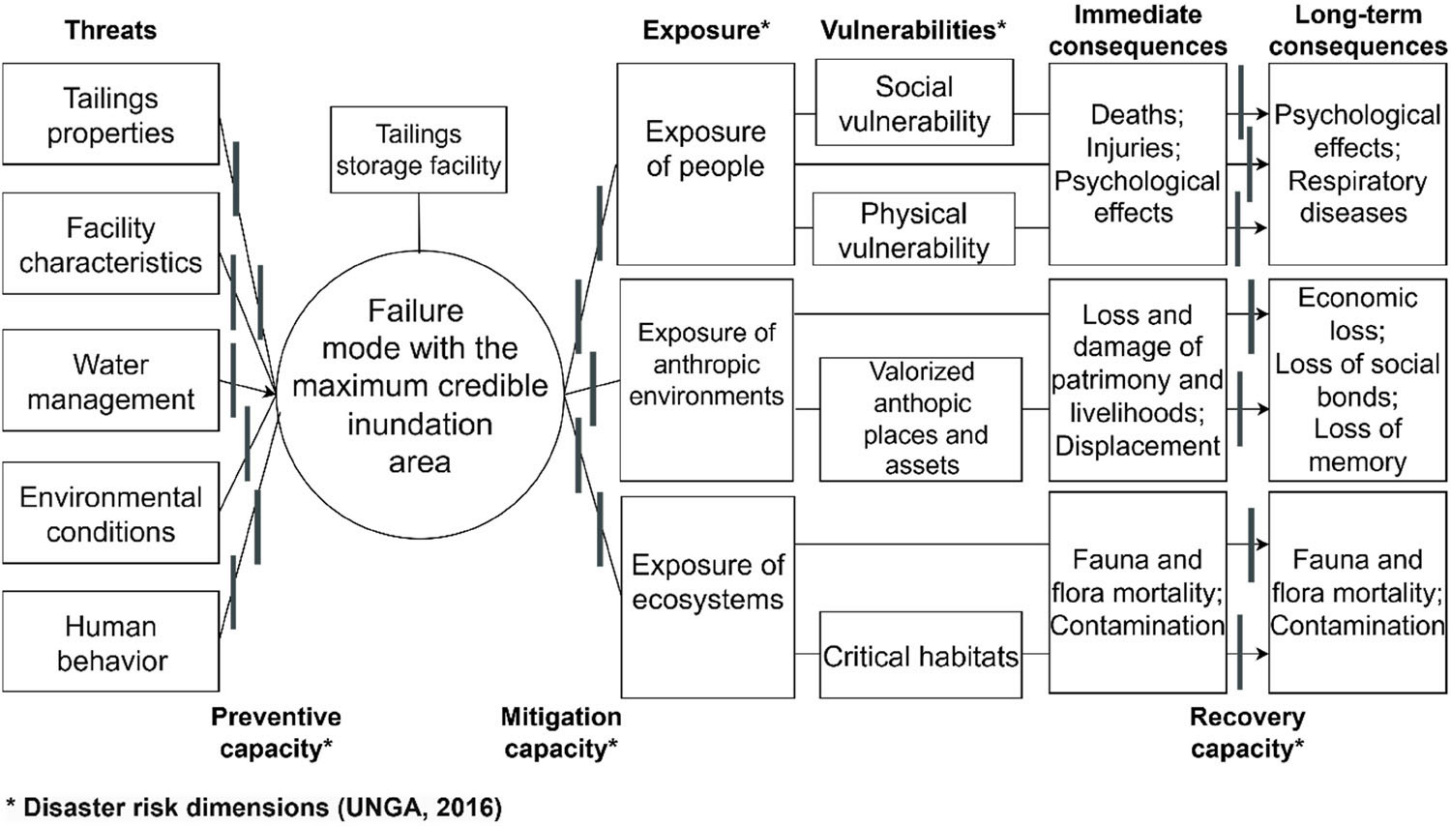
Generic adapted bow‐tie for analyzing disaster risks related to tailings storage facilities. Details are provided in Supporting Information B.

Considering exposure and vulnerability conditions, controls as capacities of disaster risks, long‐term consequences, recovery controls, and human and technical threats of TSFs’ failure, a seven‐step methodology for a TSF disaster bow‐tie is proposed in the sequence (Figure 6). Figure 7 illustrates a generic bow‐tie, showing the categories of threats, exposure, and vulnerabilities, and examples of consequences. Due to space limitations, a detailed bow‐tie is provided in Supporting Information B.

**Step 1**: Describe the worst scenario, that is, the failure mode with the maximum credible inundation area.
**Step 2**: Identify current threats. We propose 35 threats across 22 subcategories within five categories: tailings properties, facility characteristics, water management, environmental conditions, and human behavior of the company responsible for the TSF, financial institutions, and the government (Figure 7). For each subcategory that fits the TSF context, the existence of threats should be verified.
**Step 3**: Identify at least one preventive control for each threat. Controls were specified regarding six life cycle stages: (i) planning, including site investigation and TSF design development; (ii) site preparation and, in the case of dams, initial embankment construction; (iii) operation of the TSF, considering tailings disposal and, in the case of dams, raises construction; (iv) closure, when new tailings discharges cease and rehabilitation measures may be developed; (v) post‐closure, when closure activities are complete; and (6) relinquishment, when there is no company responsible for the TSF. The life cycle and control hierarchy related to each preventive control are specified in Supporting Information B.
**Step 4**: Define the (i) study area for consequences, considering the inundation area and proximities, and (ii) time‐scale of consequences and recovery capacity.
**Step 5**: Identify possible consequences. First, the exposure of people and the anthropic and natural environment should be mapped, considering the inundation study. In the sequence, social and physical vulnerabilities of people and vulnerable assets and ecosystems should be identified to enhance emergency preparedness and disaster recovery. Finally, for each exposure, at least one direct consequence—that is, caused by the wave of tailings—should be identified. In the sequence, direct consequences should be analyzed to determine whether they cause indirect immediate consequences. If long‐term consequences were scoped, they would be identified as impacts of immediate consequences. We present 35 consequences classified into secondary events, human safety, socioeconomic, and biophysical. Detailed examples of exposure and vulnerability are addressed in Section 3.1 and listed in Supporting Information B.
**Step 6**: Identify at least one mitigation control for each exposure Supporting Information B
**Step 7**: All immediate and long‐term consequences must be contemplated by recovery planning (Table B.2).

### 4.2 Case Application

**FIGURE 8:**
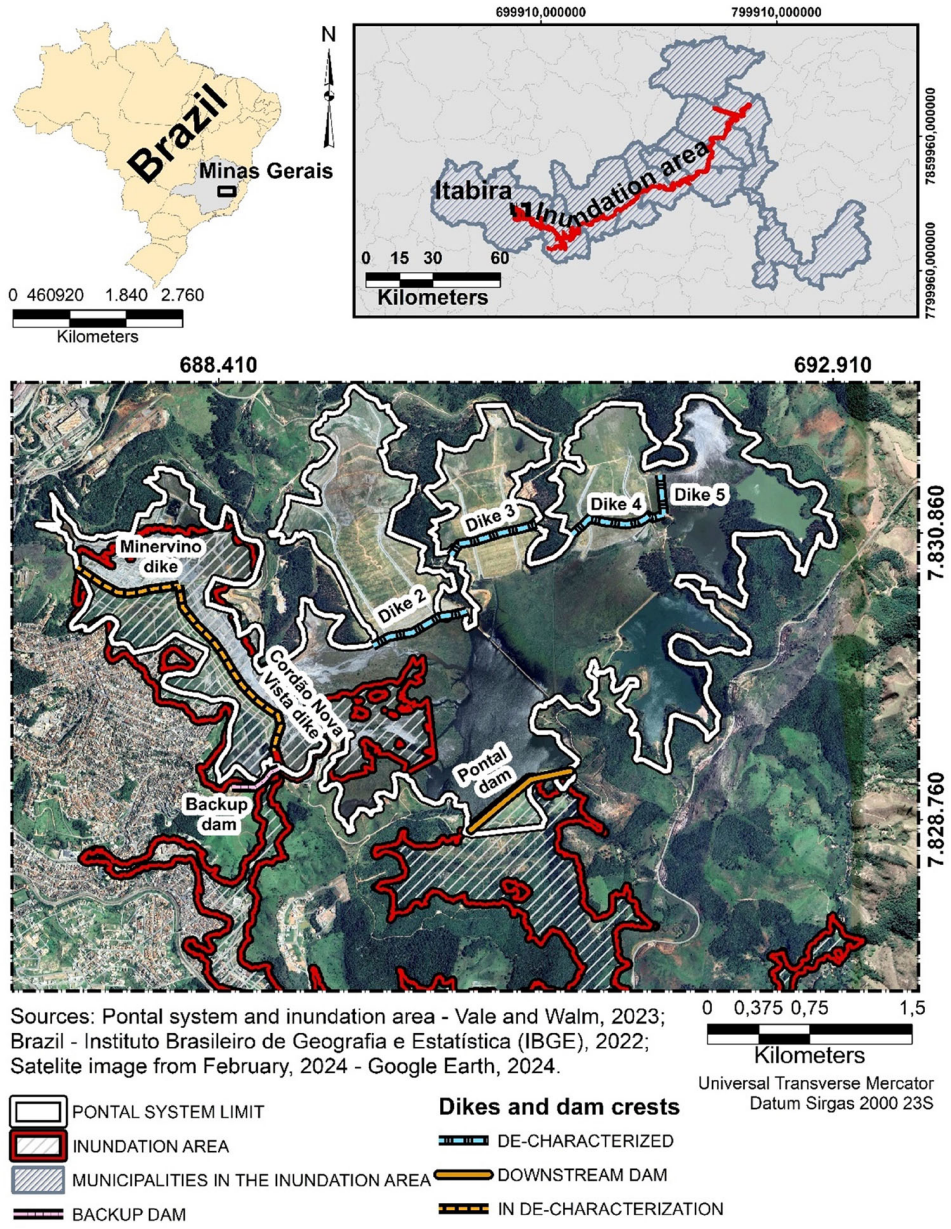
Pontal system and inundation area.

The Pontal downstream dam was constructed in 1972 in Itabira, in Minas Gerais, to store iron ore tailings from the Cauê mine, owned by Vale S. A. Since 1995, six internal upstream dikes have been constructed to increase the storage capacity, establishing the Pontal system (Figure 8). After the failure in Brumadinho, new Brazilian legislation demanded the *de‐characterization* of upstream dams, that is, decommissioning and stabilization. Therefore, the Pontal system has been in closure since 2021, and Dikes 2–5 were decommissioned. In the period, Vale (2022) reports organizational changes by implementing the GISTM, launched in 2020.

According to the emergency plan (Vale and Tetra Tech 2023), the worst failure scenario would be the breach of the Pontal dam, jointly with the Minervino and Cordão Nova Vista dikes by overtopping. To reduce the inundation area, a concrete backup dam was constructed in 2022 to retain part of the tailings in case of overtopping, resulting in an inundation extension of 185 km affecting 15 municipalities. Scoping only the inundation area for study, exposure of 26,070 people is estimated, among whom 1302 were found to have locomotion difficulties and 7169 live close to the TSF, where emergency responses from authorities are not guaranteed. As mitigation barriers, sirens are installed and emergency simulations are conducted. Furthermore, 52 legally protected tangible and intangible heritage values, as well as 10 ecological reserves, were identified. The emergency plan lists 16 watercourses that would be affected, for which there is a mitigation plan to reduce tailings dissipation.

**FIGURE 9:**
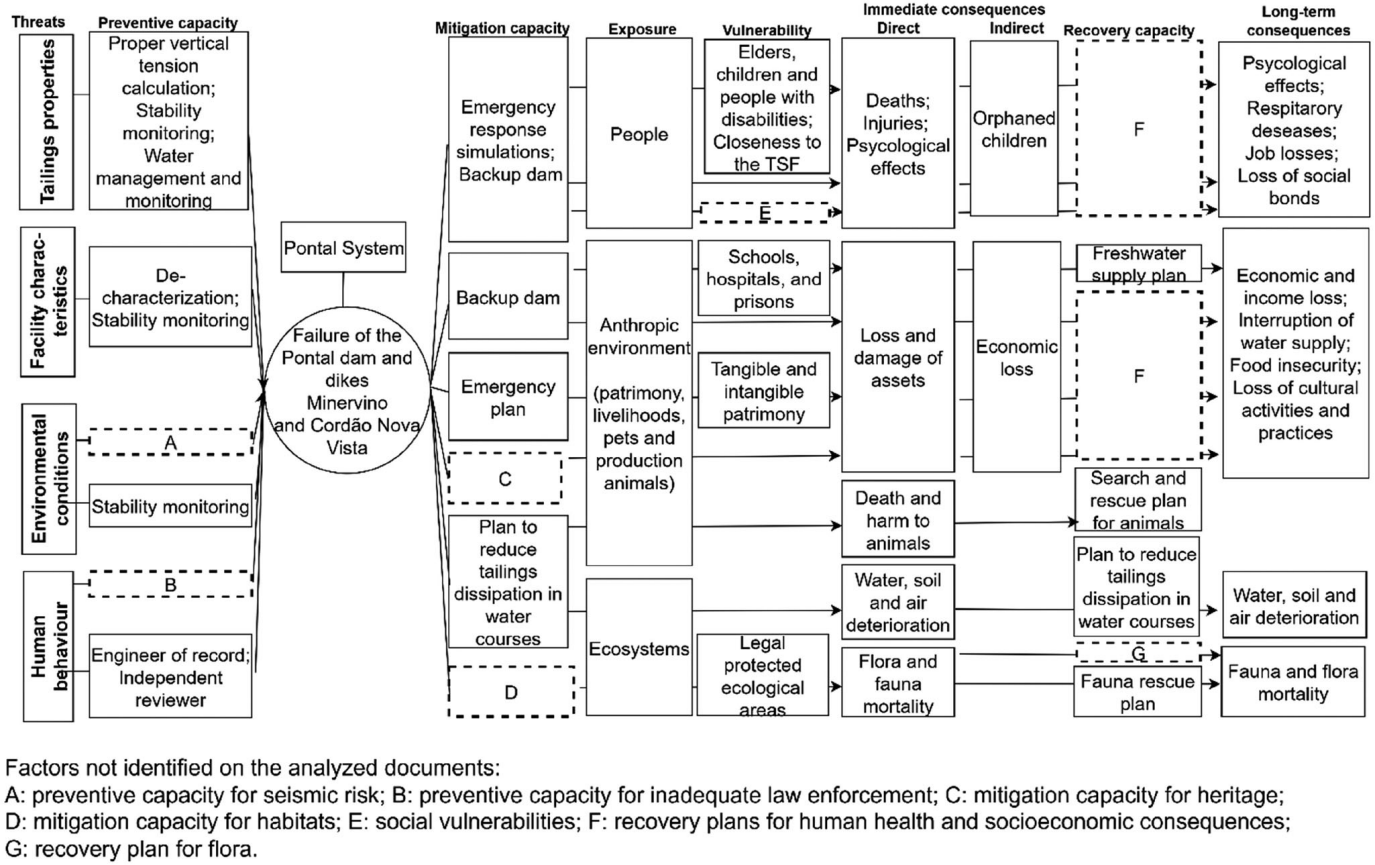
Bow‐tie framework of the disaster risks of Pontal system. TSF, tailings storage facilities.

We identified 15 threats and seven preventive controls, as illustrated in Figure 9. Due to word count limitations, the detailed references and explanations for each threat are provided in Supporting Information A. We did not identify on the analyzed documents threats of the category of water management, seismic monitoring, and governmental controls for inadequate law enforcement.

Moreover, scoping immediate and long‐term consequences, we verified 28 immediate and 20 long‐term consequences regarding 15 conditions of exposure, seven vulnerabilities, eight mitigation controls, and four recovery capacities. People's exposure is addressed on mitigation measures, whereas patrimony and habitats lack emergency response.

Inversely, recovery plans for human health and socioeconomic consequences were not identified, except for the freshwater supply plan. Only the latter recovery plan is required in advance by the Minas Gerais law (2019), in addition to economic provision for disaster recovery. GISTM also requires recovery planning only as the TSF fails (GTR 2020). These recommendations for designing in the disaster aftermath delay recovery—intensifying the consequences and challenging community participation (Milanez et al. 2021).

Social vulnerabilities were excluded from the analysis, as only non‐existent indigenous communities were identified in the documents. Notwithstanding, mapping this vulnerability is crucial to ensure the delivery of emergency preparedness information for marginalized groups, as power relations determine access to information (Kemp 2020). Moreover, these vulnerabilities are related to long‐term socioeconomic impacts, such as dependency on financial support, decreasing the resilience for disaster recovery.

In conclusion, applying the adapted bow‐tie revealed gaps in the disaster risk management of the Pontal System. For enhancement, the mining company responsible for the TSF could adopt a seismic monitoring system, implement mitigation controls to protect heritage and habitats, identify social vulnerabilities, and develop recovery plans for human health and socioeconomic consequences. Additionally, the National Mining Agency of Brazil should strengthen law enforcement by increasing independent governmental auditing to complement and counterbalance self‐reporting by mining companies.

### 4.3 Critical Assessment

Applying the proposed bow‐tie revealed which conditions of the hazard‐prone area are lacking in the emergency plan of the Pontal TSF. Although exposure and vulnerability were identified for people and natural and anthropic environments, social vulnerability was disregarded, mentioning only Indigenous people. Therefore, the framework potentially assists in identifying gaps in the disaster risk management of TSFs.

Moreover, the case application highlighted that a comprehensive risk analysis of a TSF requires diverse and ample information. This implies the need for making such information publicly available for stakeholders, other than the mining company, to conduct a risk analysis. In alignment, the Sendai Framework recommends making disaster risk information freely available (UNISDR 2015). Conversely, identification of exposure conditions could use satellite images, although identifying vulnerabilities may require fieldwork. Consequently, the framework may be more feasible for mining companies and governmental agencies, which possess extensive data about the facility and surroundings.

Although comprehensive, the proposed bow‐tie does not identify failure modes of controls. An emergency plan, for example, will only be effective if it is adequately designed and simulated. Therefore, further analysis should identify failure modes of controls and assurance requirements (Australia 2016). In addition, different threats, controls, and consequences from those listed in Supporting Information B may be applicable to TSFs, depending on their contexts. Thus, identification of factors should not be limited to our list.

In addition, the framework assists in understanding the complexity of a TSF disaster, although it does not enable calculating the risks. For risk evaluation, that is, comparing the risk level with criteria (Australia 2016), additional tools should be applied. Therefore, the adapted bow‐tie is a potential tool for identifying and integrating underlying technical, organizational, social, and environmental factors that influence disasters.

The framework also didactically separates its components into categories to facilitate their identification. Such separation does not exist in practice, as threats interact with each other, and consequences on people and the anthropic and natural environment are also related. These interactions should be considered in the risk analysis, although the proposed bow‐tie does not systematically identify them.

## 5 Conclusion

The proposed bow‐tie framework for TSF's disaster risks highlights the complexity of safely managing these facilities and the intricacy of consequences. TSF stability control is primordial and irreplaceable, although insufficient for effective disaster risk reduction. Exposure and vulnerabilities of the hazard‐prone area must be jointly considered in mitigation and recovery capacities to minimize immediate and long‐term consequences. Considering the four disaster risk dimensions will significantly improve TSF's disaster risk reduction strategies.

The applicability of the framework was proved by analyzing the Pontal system using publicly available information, made possible by recent changes in Brazilian legislation increasing access to information. We identified threats and consequences throughout the listed categories; however, not all respective preventive, mitigation, and recovery capacities were reported. Exposure and vulnerability were comprehensively documented in the emergency plan, except for social vulnerabilities. Therefore, most data required for the bow‐tie development is open access, allowing any trained person to conduct the qualitative analysis.

The mining company (TSF owner) is primarily responsible for the facility, but the framework evidences the important role of financial institutions and the government in enforcing safety. Moreover, civil society—especially people in the hazard‐prone area—must be enabled to engage in the decision‐making process about risks related to TSFs. Because TSF disaster risks tend to increase with climate change and the expected growth of critical mineral production, comprehensive disaster risk analysis must be conducted, involving multiple institutions and exposed people.

## Funding

This study was financed in part by the Coordenação de Aperfeiçoamento de Pessoal de Nível Superior—Brazil (CAPES; Finance Code 3134/33002010131P7).

## Supporting information




**Supplementary information**: risa70137‐sup‐0001‐SuppMat.docx

**Supplementary information**: risa70137‐sup‐0002‐SuppMat.docx
