# Supplementary material for: Setting a Comprehensive Bow‐Tie Framework for Disaster Risk Analysis of Mine Tailings Storage Facilities
Source: Risk Anal. 2025 Oct 28;45(12):4604–18. doi: 10.1111/risa.70137 (PMC12747679; doi:10.1111/risa.70137)
Supplement: Supplementary file 1 — Supplementary information: risa70137‐sup‐0001‐SuppMat.docx [file RISA-45-4604-s001.docx]

**Supplementary Material A – Explanations and references adopted for the Pontal study case**

On: Massignan, R. S., Siqueira-Gay, J., Sánchez, L. E. 2025. Setting a Comprehensive Bow‐Tie Framework for Disaster Risk Analysis of Mine Tailings Storage Facilities. Risk Analysis.

Supplementary Material A complements Section 4.2, which describes the Pontal case application and its results. This material is organized in two parts: firstly, the explanation and references for whether considering each component of the adapted bow-tie are presented; followed by the full list of references.

For ease of reading on A4 paper, the explanation and references for the study case are organized into four tables (according to Figure A.1). Table A.1 regards the considered threats and preventive capacities; Table A.2 explains mitigation capacities and conditions of exposure; Table A.3 presents the vulnerability conditions; and Table A.4 includes immediate consequences, recovery capacities, and long-term consequences.

**Figure A.1 – Content of Tables A.1, A.2, A.3 and A.4 presented on Supplementary Material A**


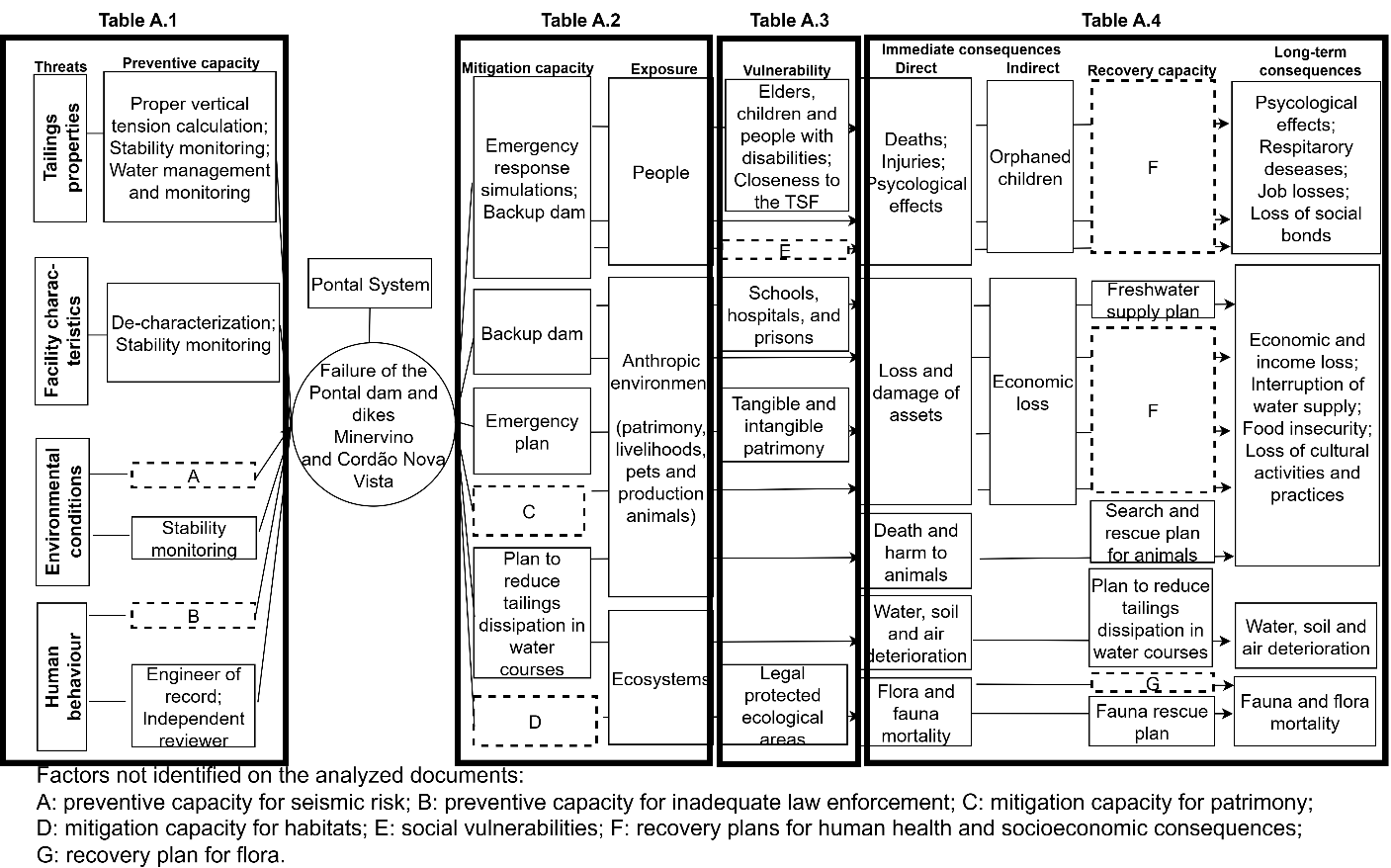


**Table A.1 – Explanations and references for each threat and preventive capacities considered on the Pontal case application. Components applicable to the Pontal case are highlighted in grey, while non-applicable components are identified by #.**

| **Category** | **Subcategory** | **Threat** | **Threat** | | **Preventive capacities** | **Preventive capacities** | | **Hierarchy level** |
| --- | --- | --- | --- | --- | --- | --- | --- | --- |
|  |  |  | **Explanation** | **References** |  | **Explanation** | **References** |  |
| **Category: Tailings properties** | **Geochemistry** | High specific density | The closure project considered the specific density of 22 kN/m^3^ for the tailings of iron ore. | Vale and Walm, 2022 | Proper vertical tensions calculation in the TSF and consideration in the TSF design development | The closure project considered the specific density of 22 kN/m^3^ for the tailings of iron ore. | Vale and Walm, 2022 | Management |
|  |  |  |  |  | Proper foundation preparation | Not applicable to the closure stage. |  |  |
|  | **Grain size distribution** | Fine particles | Size classification of tailings in the beach region of Dike 3 resulted in high percentage of clay, silt and fine sand. | Vale and Walm, 2022 | Avoiding upstream dam disposal method | Not applicable to the closure stage. |  |  |
|  |  |  |  |  | Planning grain-size distribution in dams disposal | Not applicable to the closure stage. |  |  |
|  |  |  |  |  | Dispose fine grains distantly from the dam | Not applicable to the closure stage. |  |  |
|  |  |  |  |  | Co-disposal with rock waste | Not applicable to the closure stage. |  |  |
|  | **Solid content** | Low solid content | Tailings rheology information indicate low solid content. | Vale and Tetra Tech, 2023 | Co-disposal with rock waste | Not applicable to the closure stage. |  |  |
|  |  |  |  |  | Filtering and thickening | Not applicable to the closure stage. |  |  |
|  |  |  |  |  | Avoiding upstream dam disposal method | Not applicable to the closure stage. |  |  |
|  |  |  |  |  | Water management and monitoring | Piezometers, water level indicators, flowmeter, reservoir level gauge | Vale and Tetra Tech, 2023 | Management |
| **Category: Facility characteristics** | **Construction and raise method** | #Fast raising and construction | "There are geotechnical studies that prove the degree of compaction according to the project." | ANM, 2024 | Construction and raising must be conducted according to the tailings consolidation rate | The respective threat was not identified. |  |  |
|  |  | Design is not followed | Information not found |  | Follow approved design | Information not found |  |  |
|  |  | Upstream dams | Dikes 02, Minervino, and Cordão Boa Vista were raised by upstream method and were not de-characterized. | Vale and Tetra Tech, 2023 | Downstream dam, centerline dam, dry-stack, in-pit disposal | Not applicable to the closure stage. |  | Substitution |
|  |  |  |  |  | Planning high solid tailings | Not applicable to the closure stage. |  | Engineering |
|  |  |  |  |  | Planning segregation of fine particles from the dam | Not applicable to the closure stage. |  | Engineering |
|  |  |  |  |  | Avoid tailings with less than 40% sand-sized grains | Not applicable to the closure stage. |  | Engineering |
|  |  |  |  |  | Dispose fine grains distantly from the dam | Not applicable to the closure stage. |  | Engineering |
|  |  |  |  |  | *De-characterization* | Closure aims to reduce the risk of failure. | ANM, 2024 | Elimination |
|  |  |  |  |  | Monitoring | Piezometers, water level indicators, flowmeter, reservoir level gauge, topographical marks | Vale and Tetra Tech, 2023 | Management |
|  | **Embankment** | Intermediate slope | "Intermediate (1V:2H >= Average slope gradient in the main section > 1V:3H)" | ANM, 2024 | Avoid steep slope | Not applicable to the closure stage. |  | Engineering |
|  | **Facility dimensions** | High dimensions | 218,964,640.00 m^3^ reservoir volume;  68 m height;  790 m crest length; 8,366,966 m^2^ reservoir area | ANM, 2024 | Monitoring | Piezometers, water level indicators, flowmeter, reservoir level gauge, topographical marks | Vale and Tetra Tech, 2023 | Management |
|  | **Facility age** | Advanced age | Pontal dam was constructed in 1972 | ANM, 2024 | Monitoring | The respective threat was not identified. |  |  |
|  | **Life cyle stages** | #Abandoned facilities; Inactive facilities | The Pontal TSF is in closure stage | Vale and Tetra Tech, 2023 | Monitoring | The respective threat was not identified. |  | Management |
|  |  |  |  |  | Closure planning | The respective threat was not identified. |  | Engineering |
|  |  |  |  |  | Progressive rehabilitation | The respective threat was not identified. |  | Engineering |
|  |  |  |  |  | Decommissioning and rehabilitation | The respective threat was not identified. |  | Engineering |
|  |  |  |  |  | Financial assurance and provision | The respective threat was not identified. |  | Management |
| **Category: Water management** | **Water management** | #High internal water level; Inadequate seepage; High pond level; Piping | "Percolation fully controlled by the drainage system." | ANM, 2024 | Tailings with high solid content; Beach region; Water reclamation; Spillways | The respective threat was not identified. |  | Engineering |
|  |  |  |  |  | Monitoring | The respective threat was not identified. |  | Management |
|  |  | #Overtopping | "Well-maintained civil structures in normal operation / dam without the need for spillway structures" | ANM, 2024 | Free board; Spillways | The respective threat was not identified. |  | Engineering |
|  |  |  |  |  | Adopt the largest probable flood in 10,000 years (at least) | The respective threat was not identified. |  | Management |
| **Category: Environmental conditions** | **Foundation** | #Insufficiently resistant foundation | "Foundation investigated according to project." | ANM, 2024 | Avoid foundation insufficiently resistant | The respective threat was not identified. |  | Elimination |
|  |  |  |  |  | Foundation treatment | The respective threat was not identified. |  | Engineering |
|  |  |  |  |  | Conduct studies of foundation resistance and geological layers investigation | The respective threat was not identified. |  | Engineering |
|  |  |  |  |  | Monitoring | The respective threat was not identified. |  | Management |
|  | **Seismicity** | Areas subjected to seismic activity | Although Brazil is in the middle of a tectonic plate, seisms of magnitude 5 and 6 should be considered probable to happen anywhere in the planet. | Assumpção et al., 2016 | Avoid areas subjected to seismic activity | The respective threat was not identified. |  | Elimination |
|  |  |  |  |  | Avoid upstream dam | The respective threat was not identified. |  | Engineering |
|  |  |  |  |  | Seismic activity monitoring | Information not found. |  | Management |
|  | **Mining and other technological hazards** | Rock blasting and mining accidents that may induce ground vibration | The TSF is approximately 5 km distant from an open pit. | Google Earth, 2024 | Select a construction location with an enough safety distant to rock blasting activities | Not applicable to the closure stage. |  | Engineering |
|  |  |  |  |  | Seismic activity monitoring | Information not found. |  | Management |
|  |  | #Failure of water dams, chemical industries | These installations were not identified in satellite images. | Google Earth, 2024 | Manage risks jointly with other technological hazards | The respective threat was not identified. |  | Management |
|  | **Hydrology** | #Floods | The Pontal TSF is distant from water courses. | Google Earth, 2024 | Free board; Spillways | The respective threat was not identified. |  | Engineering |
|  |  |  |  |  | Consider climate change; Adopt the largest probable flood in 10,000 years (at least) | The respective threat was not identified. |  | Management |
|  |  |  |  |  | Adaptive management | The respective threat was not identified. |  | Management |
|  | **Meteorology** | Heavy rainfall | Semi-humid equatorial climate. | IBGE, 2024. | Adaptive management | Information not found. |  | Management |
|  |  | #Heavy winds | Mean wind speed: approximately 4 m/s. | World Bank, 2017 | Consider climate change | The respective threat was not identified. |  | Management |
|  |  | Wildfire | Medium to high risk of wildfires in Itabira in June, 2024. | INPE, 2024. | Adaptive management; Emergency plan | Information not found. |  | Management |
|  | **Mass movement** | #Landslides and debris flows | The Pontal TSF is outside the mapped risk zone for erosion and mass movement. | CPRM, 2010. | Adaptive management; Emergency plan | The respective threat was not identified. |  | Management |
|  | **Climate change** | Climate change | Climate change is a global phenomenon. | IPCC, 2023 | Adaptive management; Emergency plan | The respective threat was not identified. |  | Management |
| **Category: Human behavior** | **Government responsibilities** | #Inadequate laws | After the failure in Brumadinho, TSF legislation was significantly improved, regarding federal and Minas Gerais jurisdictions. | ANM Resolution 95/2022; Minas Gerais Law 23.291/2019 | Consider international good practices on law development | The respective threat was not identified. |  | Elimination |
|  |  | Poor law enforcement | The Fundão dam and Córrego do Feijão disasters evidenced that TSFs in Brazil are majorly self-monitored by the companies, with improper State audition and law enforcement. | Rose et al., 2023 | Allocate financial, human and material resources to audit TSFs | The National Mining Agency of Brazil still lacks sufficient staff and financial resources. | Nogueira, 2023 | Management |
|  | **Investors responsibilities** | #Investment in mining companies with inadequate TSF safety management | Vale is implementing GISTM requirements. | Vale, 2022 | Disinvestment in mining companies with inadequate TSF management or TSFs failures and accidents | The respective threat was not identified. |  | Elimination |
|  |  |  |  |  | Audit TSFs for investment decisions; Enforce good practices adoption | The respective threat was not identified. |  | Management |
|  | **Laws and good practices** | Inadequate compliance with laws and safety standards | Upstream dams are not recommended by best practices. | ANM, 2024 | Engineer of record; Independent reviewer | Preventive control identified. | Vale, 2022 | Management |
|  | **Staff capacity** | #Employees not qualified | "There is an administrative unit with a qualified technical professional responsible for the safety of the dam or it is a dam not classified under items I, II, III or IV, sole paragraph of art. 1 of Law No. 12,334/2010." | ANM, 2024 | Hire qualified employers | The respective threat was not identified. |  | Elimination |
|  |  |  |  |  | Offer qualifying courses and encourage employers to take new courses |  |  | Management |
|  | **Internal communication** | #Employees are not motivated to report safety issues of the TSF | "Establishing mechanisms to recognize, reward and protect employees who identify and report opportunities for improvement in TSF management" | Vale, 2022 | Mechanisms for anonymous complaints; Develop safety culture | There is no evidence of significant change in the State auditing of TSFs. |  | Management |
|  |  | #Inexistent or inadequate procedures of change management | "Executive project or "as built" | ANM, 2024 | Update failure risk analysis considering changes; Documentation of the TSF | There is no evidence of significant change in the State auditioning of TSFs. |  | Management |
|  | **Financial resources** | Profit or other goals is prioritized over safety in decision making | This threat was identified in the Dam 1 failure, in Brumadinho, in 2019. Since changes in human behavior are not immediate, this threat was still considered. | Hopkins and Kemp, 2021 | Full cost accounting; Director panelists and CEO are accountable for TSF’s failures | No evidence was found by the authors |  |  |
|  |  |  |  |  | Engineer responsible for the TSF responds to the responsible executive | Preventive control identified. | Vale, 2022 | Management |
|  |  | #Bonus for employees do not consider incentives related to safety | "The Safety and Operational Excellence area does not have a goal linked to production or financial metrics" | Vale, 2022 | Develop safety culture | The respective threat was not identified. |  | Management |

**Table A.2 – Explanations and references for each mitigation capacity and exposure condition considered on the Pontal case application. Components applicable to the Pontal case are highlighted in grey, while non-applicable components are identified by #.**

| **Mitigation capacities** | **Mitigation capacities** | | **Exposure** | | **Exposure** | |
| --- | --- | --- | --- | --- | --- | --- |
|  | **Explanation** | **Reference** |  |  | **Explanation** | **Reference** |
| #Reduce people exposure by zoning regulations and land use policies | People in the inundation area were not displaced. | Vale and Tetra Tech, 2023 | **People** | Mine staff; Residents; Passerby, tourists | About 26,070 people were identified in the inundation area. | Vale and Civil Defense of Itabira, 2023 |
| Emergency response simulations;  Consider vulnerabilities in emergency plan; Risk communication; Sirens, escape routes, signs, and meeting places for emergencies | Mitigation capacities identified. | Vale and Itabira Civil Defense, 2023; Vale and Tetra Tech, 2023 |  |  |  |  |
| #Reduce anthropic exposure | Applicable to planning stage. |  | **Anthropic environment** | Constructed houses and buildings (residences, work places, etc.) | Exposure of constructed houses and buildings is identified. | Vale and Tetra Tech, 2023 |
|  |  |  |  | Water and effluent treatment installations | 7 water treatment installations and 9 effluent treatment installations. | Vale and Civil Defense of Itabira, 2023 |
| Containment walls | Backup dam Coqueirinho aims to reduce the inundation area in case of failure of the Dikes Minervino and Cordão Nova Vista | Vale and Tetra Tech, 2023 |  | Leisure areas and establishments | 38 leisure areas and establishments (e.g. public squares, gyms) | Vale and Civil Defense of Itabira, 2023 |
|  |  |  |  | Mobile assets | Although not mapped in the Emergency plan, it was assumed that exposed people own mobile assets. |  |
|  |  |  |  | Railways, accesses | Exposure of railways and accesses were identified. | Vale and Tetra Tech 2023 |
|  |  |  |  | Croplands, exposed soil | Exposure of croplands and exposed soil were identified. | Vale, 2023a |
| Consider vulnerabilities in emergency plan - heritage rescue plan. | Patrimony with legal instruments of protection were identified: 36 'inventários' (tangible and intangible patrimony under identification, research, documentation, and management), 11 'registros' (intangible patrimony), 5 'tombamento' (tangible patrimony) | Vale and Estilo Nacional, 2021 |  | Pets, captive wild animals, and production animals | Exposure of pets, captive wild animals, and production animals was identified. | Vale, 2023a; Vale, 2023b |
|  |  |  |  | Energy production infrastructure (including hydropower installations) | Not identified in the Emergency Plan. | Vale and Civil Defense of Itabira, 2023 |
|  |  |  |  | #Industrial facilities | Not identified in the Emergency Plan. | Vale and Civil Defense of Itabira, 2023 |
| #Reduce natural environment exposure | Applicable to planning stage. |  | **Natural environment** | Habitats (including water courses) | Areas of native vegetataion;  Water courses - Córrego do Periquito, Córrego dos Doze, Ribeirão do Peixe, Córrego do Cachoeira, Córrego Santa Cruz, Córrego Goiabeira, Ribeirão São José, Córrego do Apaga Pito, Rio Piracicaba, Ribeirão Piçarrão, Córrego Indaiá, Ribeirão Grande, Rio Doce, Rio Taquaraçu, Rio Branco, Rio Santo Antônio | Vale and Tetra Tech, 2023; Vale and Civil Defense of Itabira, 2023 |
| #Fauna and flora rescue plan | There is only rescue plan for domestic and exotic pets and production animals (considered in the anthropic environment) | Vale, 2023 |  | Fauna | Although not mapped in the Emergency plan, it was considered the fauna of exposed habitats. |  |
| Containment walls and nets, in case of water bodies. | There is a mitigation plan to reduce tailings dissipation. | Vale and HidroBR, 2023 |  | Flora | Although not mapped in the Emergency plan, it was considered the flora of exposed habitats. |  |

**Table A.3 – Explanations and references for each vulnerability considered on the Pontal case application. Components applicable to the Pontal case are highlighted in grey, while non-applicable components are identified by #.**

| **Exposure** | **Vulnerability** | | **Vulnerability** | | | |
| --- | --- | --- | --- | --- | --- | --- |
|  |  |  | **Explanation** | | **Reference** | |
| **People** | **Physical vulnerability of people** | Elders, children or disable people;  Closeness to the TSF, without proper time for emergency response. | 1,302 people have locomotion difficulties;  7,169 people are within 10 km of the inundation area, where emergency response from authorities is not guaranteed, due to lack of proper time. | Vale and Itabira Civil Defense, 2023 | |  |
|  | **Social** | #Indigenous communities | Indigenous communities will not be affected in case of dam failure. | Vale and Tetra Tech, 2023. | |  |
|  |  | Low-income people; Excluded groups due to race and language; Illiterate | No information mapped in the Emergency Plan. |  | |  |
| **Anthropic environment** | **Impose physical vulnerability on people** | Schools, hospitals and prisons | 5 Schools, 11 hospitals, and 5 prisons were identified. | Vale and Itabira Civil Defense, 2023 | |  |
|  | **Valorized anthropic places and assets** | Tangible and intangible heritage;  Valorized spiritual places | Patrimony with legal instruments of protection were identified: 36 'inventários' (tangible and intangible patrimony under identification, research, documentation, and management), 11 'registros' (intangible patrimony), 5 'tombamento' (tangible patrimony) | Vale and Estilo Nacional, 2021 | |  |
|  |  | #Archeological sites | Not identified | Vale and Estilo Nacional, 2021 | |  |
| **Natural environment** | **Vulnerable ecosystems** | Habitats with endangered, endemic and/or migratory species; Ecosystems that provide relevant ecosystem services; Highly endangered and/or unique ecosystems; Regions with high biodiversity; Valorized places for their natural beauty. | Ecological reserves: APA Municipal Antônio  Dias, APA Municipal Belo Oriente, APA Municipal Nova Era, APA Municipal Lagoa Silvana, APA Municipal Piracicaba, APA Municipal Pureza, APA Municipal Santana do Paraíso, Parque Estadual do Rio Doce, RPP Guilman Amorim, RPPN Vila Ana Angelica | Vale, 2023a | |  |

**Table A.4 – Explanations and references for each recovery capacity, considered on the Pontal case application. Components applicable to the Pontal case are highlighted in grey, while non-applicable components are identified by #.**

| **Exposure** | **Immediate consequences** | | | **Recovery capacities** | **Recovery capacities** | | **Medium and long-term consequences** |
| --- | --- | --- | --- | --- | --- | --- | --- |
|  |  | **Direct** | **Indirect** |  | **Explanation** | **References** | **Indirect** |
| **People** | **Human health** | Deaths; Injuries; Psychological effects (emotional instability, distress); |  | #Psychological support; Health monitoring and assistance | Not identified. |  | Psychological effects (emotional instability, distress, anxiety, depression); Increase in alcohol and drugs;  Increase of unrest and fear in neighboring communities who are exposed to tailings risks; Respiratory diseases |
|  | **Socioeconomic** |  | Orphaned children | #Financial compensation; Resettlement | Not identified. |  | Job loss;  Increase in criminality; Dependency of affected people for financial support; Loss of social bonds (changing friendships and family structures);  Loss of cultural activities and practices |
| **Anthropic environment** |  | Loss and damage of buildings (residences, work places, etc.) | Displacement; Loss of income; Public and private economic loss; Interruption of public services (e.g. hospitals, schools); Interruption of energy and water supply; Interruption of communication; Damage to natural livelihoods | #Reconstruction | Not identified. |  | Loss of tourism; Loss of social bonds; Loss of memory (e.g. photos). |
|  |  | Loss and damage of mobile asset |  | #Reconstruction | Not identified. |  |  |
|  |  | Loss and damage of energy infrastructure |  | #Reconstruction | Not identified. |  |  |
|  |  | Loss and damage of transport infrastructure |  | #Reconstruction | Not identified. |  |  |
|  |  | Damage of livelihoods |  | #Recovery and remediation of livelihoods | Freshwater supply plan | Vale and Arcadis, 2021; Vale and Arcadis, 2023. |  |
|  |  | Death and injuries of pets and production animals |  | Veterinarian assistance to pets and production animals | Search and rescue plan for pets, wild captive animals, and production animals. | Vale, 2023a; Vale, 2023b; Vale, 2023c |  |
|  |  | Loss and damage of intangible patrimony |  | #Recovery of patrimony | Not identified. |  |  |
|  | **Secondary events** | Failure of water dams | Increase of the inundation area | #Reconstruction; Recovery and remediation of contaminated areas | Not identified. |  | - |
|  |  | Release of toxic material | Addition of toxic material to the tailings wave |  | Not identified. |  | - |
| **Natural environment** | **Biophysical** | Water, soil and air deterioration; Sediments | - | Recovery and remediation of contaminated areas; Veterinarian assistance to injured animals | Applicable to planning stage. |  | Fauna and flora mortality; Increase in mosquito-borne diseases; Water and air contamination |
|  |  | Fauna mortality and injuries | - |  | Fauna rescue plan | Vale, 2023a |  |
|  |  | Flora mortality | - |  | There is a mitigation plan to reduce tailings dissipation. | Vale and HidroBR, 2023 |  |

**References**

ANM, 2024. Integrated Management System of Mining Dams (SIGBM). Available in: https://app.anm.gov.br/SIGBM/BarragemPublico/Detalhar/C27FEA5A92DD903A41245C1585E4CD36A3E25443A151F77B3EB03E37EBD1F0FB . Access in: 31 July, 2024.

Assumção, M., Pirchiner, M., Dourado, J. C., Barros, L. V., 2016. Earthquakes in Brazil: preparing for rare events. Boletim SBGF, 96. https://sbgf.org.br/noticias/images/Boletim_96-2016.pdf

CPRM, 2010. Geodiverse map of the State of Minas Gerais. Available in: https://idesisema.meioambiente.mg.gov.br/geonetwork/srv/api/records/12e3e3c1-a9ab-4e39-8e12-badcd81e43df . Access in 31 July, 2024.

Google Earth, 2024. satellite image of Itabira dated from 27 February, 2024. IBGE, 2024. Brazil - Physical and Environmental Maps. Available in: https://mapasinterativos.ibge.gov.br/sigibge/ . Access in 31 July, 2024.

INPE, 2024. BDQueimadas. Available in: https://terrabrasilis.dpi.inpe.br/queimadas/bdqueimadas/#mapa . Access in 31 July, 2024.

IPCC, 2023. Climate Change 2023: Synthesis Report. Contribution of Working Groups I, II and III to the Sixth Assessment Report of the Intergovernmental Panel on Climate Change [Core Writing Team, H. Lee and J. Romero (eds.)]. IPCC, Geneva, Switzerland, 184 pp., doi: 10.59327/IPCC/AR6-9789291691647.

Nogueira, M., 2023. During general strike, ANM delays transfers of more than R$1 billion to municipalities, says association. Uol, 29 August, 2023. Available in: https://noticias.uol.com.br/ultimas-noticias/reuters/2023/08/29/em-greve-geral-anm-atrasa-repasses-de-mais-de-r1-bi-a-municipios-diz-associacao.htm . Access in 01 Augst 2024.

Rose, R.L., Mugi, S.R., Saleh, J.H., 2023. Accident investigation and lessons not learned: AcciMap analysis of successive tailings dam collapses in Brazil. Reliab Eng Syst Saf 236, 109308. https://doi.org/10.1016/j.ress.2023.109308 Vale, 2022. Vale ESG Webinar: dams and tailings management. 25 March , 2022. Available in: https://vale.com/documents/44618/1074926/25.03.2022+-+Vale+ESG+Webinar+Gest%C3%A3o+de+barragens+e+rejeitos.pdf/4f366749-de85-a463-ae18-b579268f320a?version=1.1&t=1696884088934&download=false . Access in 31 July, 2024.

Vale, Walm Engenharia, 2022. Pontal dam de-characterization project - Dike 3 - detailed project. Available in: https://barragens.mpmg.mp.br/diques-da-barragem-do-sistema-pontal/ . Access in 31 July, 2024.

Vale, Walm Engenharia, 2023. Pontal dam de-characterization project - Dike 2 - detailed project. Available in: https://barragens.mpmg.mp.br/diques-da-barragem-do-sistema-pontal/ . Access in 31 July, 2024.

World Bank, 2017. Global Wind Atlas, “World wind speed and wind power potential maps”. Available in: https://globalwindatlas.info/en/ . Access in 31 July, 2024.

**Chapters from the Pontal TSF Emergency plan (PAEBM) - Avaible in: https://vale.com/pt/paebm . access in 31 July, 2024**

Vale, 2023a. Action plan for rescuing wild and exotic fauna, captive and free-lived, in emergency situations and dam breakage.

Vale, 2023b. Evacuation, rescue, salvage and destination plan for domestic fauna in an emergency situation and break of the system pontal dam.

Vale, 2023c. Action plan for the preservation and safeguarding production animals.

Vale, Arcadis, 2021. Diagnostic of the current situation and alternative solutions for public systems of supply and alternative solutions to affected consumers of municipalities impacted by the Pontal System inundation area.

Vale, Arcadis, 2023. Chapter 2 – Plan of freshwater supply – Pontal System.

Vale, HidroBR, 2023. Mitigation plan for tailings leakage.

Vale, Tetra Tech, 2023. Pontal system Tailings Dam Emergency Plan (PAEBM).

Vale, Itabira Civil Defense, 2023. Civil defense and protection actions and drinking water supply plan.

Vale, Estilo Nacional, 2021. Emergency plan for the cultural patrimony.
