# Supplementary material for: Setting a Comprehensive Bow‐Tie Framework for Disaster Risk Analysis of Mine Tailings Storage Facilities
Source: Risk Anal. 2025 Oct 28;45(12):4604–18. doi: 10.1111/risa.70137 (PMC12747679; doi:10.1111/risa.70137)
Supplement: Supplementary file 2 — Supplementary information: risa70137‐sup‐0002‐SuppMat.docx [file RISA-45-4604-s002.docx]

**Supplementary Material B – Non-exhaustive list of components of the adapted bow-tie diagram for tailings storage facilities**

On: Massignan, R. S., Siqueira-Gay, J., Sánchez, L. E. 2025. Setting a Comprehensive Bow‐Tie Framework for Disaster Risk Analysis of Mine Tailings Storage Facilities. Risk Analysis.

Supplementary Material B provides a systematic listing of the components of the adapted bow-tie diagram, which are described in Section 3. This Supplementary Material also complements Figure 9 of the article, which, due to space limitation, do not include the full set of components.

For ease of reading on A4 paper, the diagram elements are organized into two tables (Figure B.1). Table B.1 presents a non-exhaustive list of threats, along with their categories and subcategories, as well as preventive capacities, categorized by life cycle stage and control hierarchy. Table B.2 includes a non-exhaustive list of mitigation capacities, conditions of exposure and vulnerability, immediate consequences, recovery capacities, and long-term consequences.

**Figure B.1 – Content of Tables B.1 and B.2 presented on Supplementary Material B**


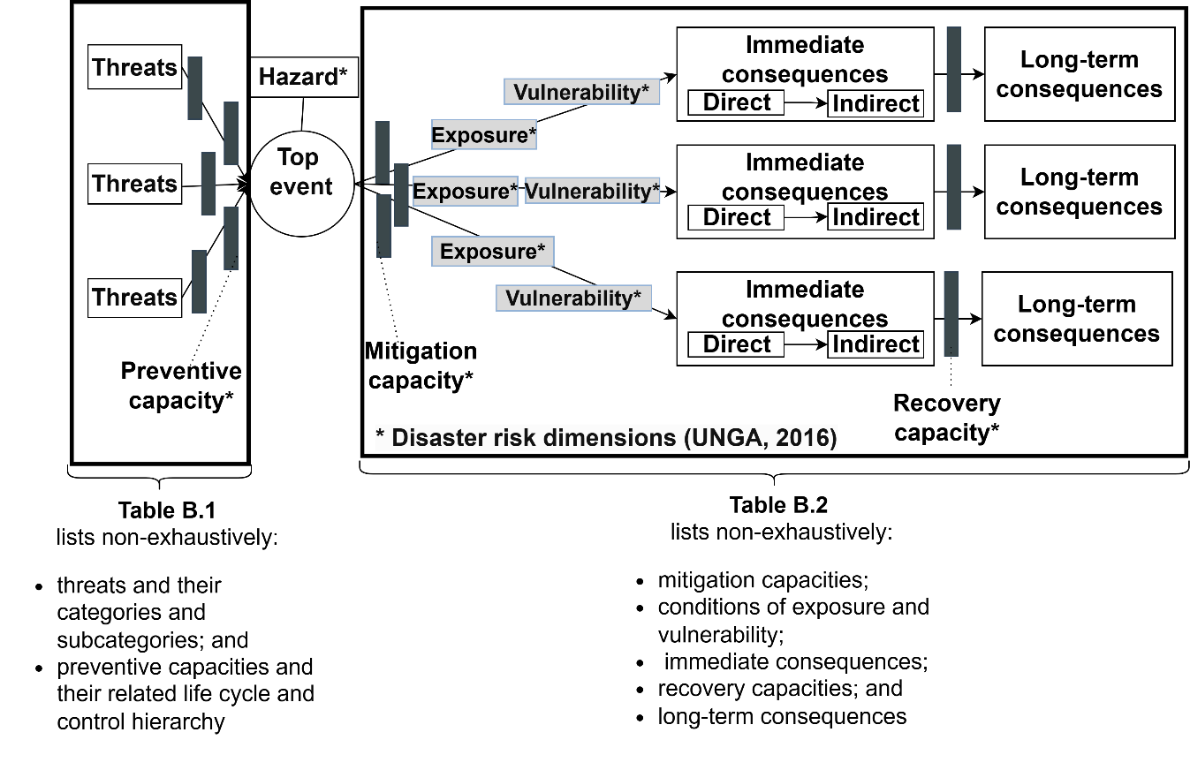


**Table B.1 – Non-exhaustive list of threats and preventive capacities for tailings storage facility (TSF) failures**

|  | **Subcategory** | **Threat** | **Preventive capacities** | **TSF life cycle** | **Hierarchy level** |
| --- | --- | --- | --- | --- | --- |
| **Category: Tailings properties** | **Geochemistry** | High specific density | Proper vertical tensions calculation in the TSF and consideration in the TSF design development | Planning, operation, closure, post-closure, abandonment | Management |
|  |  |  | Proper foundation preparation | Construction | Engineering |
|  | **Grain size distribution** | Fine particles | Avoiding upstream dam disposal method | Planning | Engineering |
|  |  |  | Planning grain-size distribution in dams disposal | Planning | Engineering |
|  |  |  | Dispose fine grains distantly from the dam | Operation | Engineering |
|  |  |  | Co-disposal with rock waste | Planning, operation | Engineering |
|  | **Solid content** | Low solid content | Co-disposal with rock waste | Planning, operation | Engineering |
|  |  |  | Filtering and thickening | Planning, operation | Substitution |
|  |  |  | Avoiding upstream dam disposal method | Planning | Engineering |
|  |  |  | Water management and monitoring | Construction, operation, closure, post-closure, abandonment | Management |
| **Category: Facility characteristics** | **Construction and raise method** | Fast raising and construction | Construction and raising must be conducted according to the tailings consolidation rate | Operation | Engineering |
|  |  | Design is not followed | Follow approved design | Construction, operation, closure | Elimination |
|  |  | Upstream dams | Downstream dam, centerline dam, dry-stack, in-pit disposal | Planning | Substitution |
|  |  |  | Planning high solid tailings | Planning | Engineering |
|  |  |  | Planning segregation of fine particles from the dam | Planning | Engineering |
|  |  |  | Avoid tailings with less than 40% sand-sized grains | Operation | Engineering |
|  |  |  | Dispose fine grains distantly from the dam | Operation | Engineering |
|  |  |  | Monitoring | Operation, closure, post-closure, abandonment | Management |
|  | **Embankment** | Steep slope | Avoid steep slope | Planning, construction, operation | Engineering |
|  | **Facility dimensions** | High dimensions | Monitoring | Operation, closure, post-closure, abandonment | Management |
|  | **Facility age** | Advanced age | Monitoring |  |  |
|  | **Life cycle stages** | Abandoned facilities; Inactive facilities | Monitoring | Operation, closure, post-closure, abandonment | Management |
|  |  |  | Closure planning | Planning | Engineering |
|  |  |  | Progressive rehabilitation | Operation | Engineering |
|  |  |  | Decommissioning and rehabilitation | Closure, abandonment | Engineering |
|  |  |  | Financial assurance and provision | Planning | Management |
| **Category: Water management** | **Water management** | High internal water level; Inadequate seepage; High pond level; Piping | Tailings with high solid content; Beach region; Water reclamation; Spillways | Planning, operation, closure | Engineering |
|  |  |  | Monitoring | Operation, operation, closure, post-closure, abandonment | Management |
|  |  | Overtopping | Free board; Spillways | Planning, operation, closure | Engineering |
|  |  |  | Adopt the largest probable flood in 10,000 years (at least) | Planning | Management |
| **Category: Environmental conditions** | **Foundation** | Insufficiently resistant foundation | Avoid foundation insufficiently resistant | Planning, construction | Elimination |
|  |  |  | Foundation treatment | Planning, construction | Engineering |
|  |  |  | Conduct studies of foundation resistance and geological layers investigation | Planning | Engineering |
|  |  |  | Monitoring | Operation, closure, post-closure, abandonment | Management |
|  | **Seismicity** | Areas subjected to seismic activity | Avoid areas subjected to seismic activity | Planning | Elimination |
|  |  |  | Avoid upstream dam | Planning | Engineering |
|  |  |  | Seismic activity monitoring | Construction, operation, closure, post-closure, abandonment | Management |
|  | **Mining and other technological hazards** | Rock blasting and mining accidents that may induce ground vibration | Select a construction location with an enough safety distant to rock blasting activities | Planning | Engineering |
|  |  |  | Seismic activity monitoring | Construction, operation, closure, post-closure, abandonment | Management |
|  |  | Failure of water dams, chemical industries | Manage risks jointly with other technological hazards | Construction, operation, closure, post-closure, abandonment | Management |
|  | **Hydrology** | Floods | Free board; Spillways | Construction, operation, closure, post-closure, abandonment | Engineering |
|  |  |  | Consider climate change; Adopt the largest probable flood in 10,000 years (at least) | Planning | Management |
|  |  |  | Adaptive management | Construction, operation, closure, post-closure, abandonment | Management |
|  | **Meteorology** | Heavy rainfall | Adaptive management | Construction, operation, closure, post-closure, abandonment | Management |
|  |  | Heavy winds | Consider climate change | Planning | Management |
|  |  | Wildfire | Adaptive management; Emergency plan | Construction, operation, closure, post-closure, abandonment | Management |
|  | **Mass movement** | Landslides and debris flows | Adaptive management; Emergency plan | All life cycle stages | Management |
|  | **Climate change** | Climate change | Adaptive management; Emergency plan | All life cycle stages | Management |
| **Category: Human behavior** | **Government responsibilities** | Inadequate laws | Consider international good practices on law development | All life cycle stages | Elimination |
|  |  | Poor law enforcement;  Active corruption | Allocate financial, human and material resources to audit TSFs | All life cycle stages | Management |
|  | **Investors responsibilities** | Investment in mining companies with inadequate TSF safety management | Disinvestment in mining companies with inadequate TSF management or TSFs failures and accidents | All life cycle stages | Elimination |
|  |  |  | Audit TSFs for investment decisions; Enforce good practices adoption | All life cycle stages | Management |
|  | **Laws and good practices** | Inadequate compliance with laws and safety standards; Active corruption;  Document falsification | Engineer of record; Independent reviewer | All life cycle stages | Management |
|  | **Staff capacity** | Employees not qualified | Hire qualified employers | All life cycle stages | Elimination |
|  |  |  | Offer qualifying courses and encourage employers to take new courses | All life cycle stages | Management |
|  | **Internal communication** | Employees are not motivated to report safety issues of the TSF | Mechanisms for anonymous complaints; Develop safety culture | All life cycle stages | Management |
|  |  | Inexistent or inadequate procedures of change management | Update failure risk analysis considering changes; Documentation of the TSF | All life cycle stages | Management |
|  | **Financial resources** | Profit or other goals is prioritized over safety in decision making | Full cost accounting; Director panelists and CEO are accountable for TSF’s failures; Engineer responsible for the TSF responds to the responsible executive | All life cycle stages | Management |
|  |  | Bonus for employees do not consider incentives related to safety | Develop safety culture | All life cycle stages | Management |

**Table B.2 – Non-exhaustive list of consequences and mitigation and recovery capacities related to tailings storage facility (TSF) failures**

| **Mitigation capacities** | **Exposure** | | **Vulnerability** | | **Immediate consequences** | | | **Recovery capacities** | **Medium and long-term consequences** |
| --- | --- | --- | --- | --- | --- | --- | --- | --- | --- |
|  |  |  |  |  |  | **Direct** | **Indirect** |  | **Indirect** |
| Reduce people exposure by zoning regulations and land use policies; Emergency response simulations; Consider vulnerabilities in emergency plan; Risk communication; Sirens, scape routes, signs, and meeting places for emergencies | **People** | Mine staff; Residents; Passerby, tourists | **Physical vulnerability of people** | Elders, children or disable people; Closeness to the TSF, without proper time for emergency response. | **Human health** | Deaths; Injuries; Psychological effects (emotional instability, distress) |  | Psychological support; Health monitoring and assistance | Psychological effects (emotional instability, distress, anxiety, depression);  Increase in alcohol and drugs;  Increase of unrest and fear in neighboring communities who are exposed to tailings risks; Respiratory diseases |
|  |  |  | **Social** | Low-income people; Indigenous communities; Women; Excluded groups due to race and language; Illiterate | **Socioeconomic** |  | Orphaned children | Financial compensation; Resettlement | Job loss;  Increase in criminality; Dependency of affected people for financial support; Loss of social bonds;  Loss of cultural activities and practices |
| Reduce anthropic exposure; Containment walls; Consider vulnerabilities in emergency plan - patrimony rescue plan. | **Anthropic environment** | Constructed houses and buildings (residences, work places, etc.) | **Impose physical vulnerability on people** | Schools, hospitals and prisons |  | Loss and damage of buildings (residences, work places, etc.) | Displacement; Loss of income; Public and private economic loss; Interruption of public services (e.g. hospitals, schools); Interruption of energy and water supply; Interruption of communication; Damage to natural livelihoods | Reconstruction; Recovery and remediation of livelihoods; Recovery of patrimony; Freshwater supply; Veterinarian assistance to pets and production animals | Loss of tourism; Loss of social bonds; Loss of memory (e.g. photos). |
|  |  | Mobile assets |  |  |  | Loss and damage of mobile asset |  |  |  |
|  |  |  | **Valorized anthropic places and assets** | Tangible and intangible heritage; Valorized spiritual places; Archeological sites. |  | Loss and damage of energy infrastructure |  |  |  |
|  |  | Energy infrastructure |  |  |  | Loss and damage of transport infrastructure |  |  |  |
|  |  | Transport infrastructure |  |  |  | Damage of livelihoods |  |  |  |
|  |  | Croplands |  |  |  | Death and injuries of pets and production animals |  |  |  |
|  |  | Pets and production animals |  |  |  | Loss and damage of intangible patrimony |  |  |  |
|  |  | Water dams | - | - | **Secondary events** | Failure of water dams | Increase of the inundation area | Reconstruction; Recovery and remediation of contaminated areas | - |
|  |  | Industrial facilities | - | - |  | Release of toxic material | Addition of toxic material to the tailings wave |  | - |
| Reduce natural environment exposure; Fauna and flora rescue plan; Containment walls and nets, in case of water bodies. | **Natural environment** | Habitats (including water courses) | **Vulnerable ecosystems** | Habitats with endangered, endemic and/or migratory species; Ecosystems that provide relevant ecosystem services; Highly endangered and/or unique ecosystems; Regions with high biodiversity; Valorized places for their natural beauty. | **Biophysical** | Water, soil and air deterioration; Sediments | - | Recovery and remediation of contaminated areas; Veterinarian assistance to injured animals | Fauna and flora mortality; Increase in mosquito-borne diseases; Water and air contamination |
|  |  | Fauna |  |  |  | Fauna mortality and injuries | - |  |  |
|  |  | Flora |  |  |  | Flora mortality | - |  |  |
